# Supplementary material for: CNS myelination requires VAMP2/3-mediated membrane expansion in oligodendrocytes
Source: Nat Commun. 2022 Sep 23;13:5583. doi: 10.1038/s41467-022-33200-4 (PMC9508103; doi:10.1038/s41467-022-33200-4)
Supplement: Supplementary file 1 — Supplementary Information [file 41467_2022_33200_MOESM1_ESM.pdf]

Supplementary Fig. 1

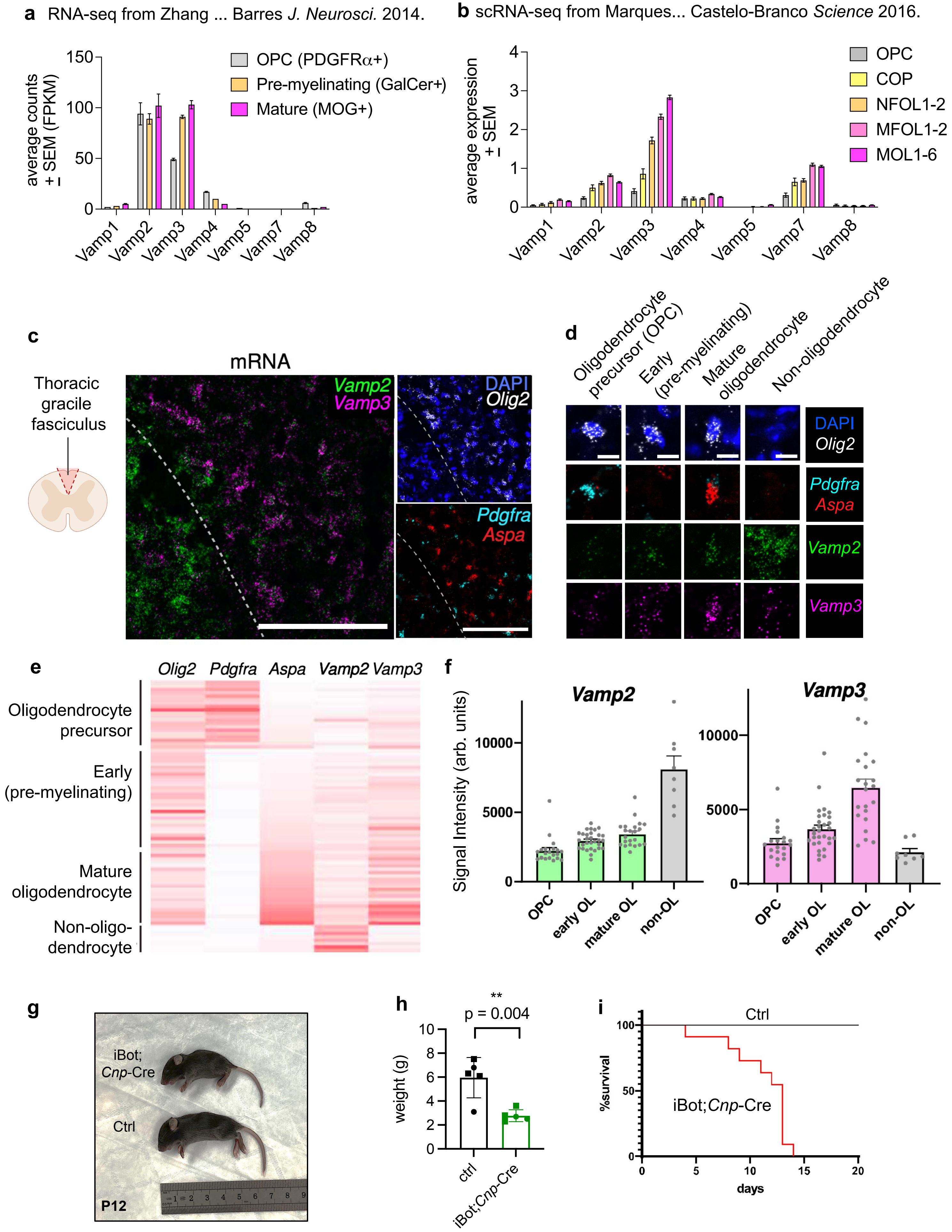

Supplementary Fig. 1 | Expression of VAMP isoforms in myelinating oligodendrocytes.

(**a-b**) Expression of v-SNAREs (*Vamp*) in oligodendrocyte-lineage cells from published data sets. (a) Replotted RNA-seq analysis from Zhang *et al. J. Neurosci.* 2014[1] with PDGFRα+ to identify oligodendrocyte precursors (OPC), GalCer+ to identify pre-myelinating, and MOG+ to mark mature oligodendrocytes. (b) Replotted single cell RNA-seq (scRNA-seq) analysis from Marques *et al*. *Science* 2016[2], where OPC = oligodendrocyte precursors; COP = differentiation-committed oligodendrocyte precursors; NFOL = newly formed oligodendrocytes; MFOL = myelin-forming oligodendrocytes; MOL = mature oligodendrocytes.

(**c**) Fluorescent *in situ* hybridization (RNAscope) of P10 wild-type mouse spinal cord cross section with probes against the mRNAs *Vamp2*, *Vamp3*, *Olig2, Pdgfra* and *Aspa*. Similar results were obtained between two spinal cord regions from one mouse. Dotted lines show the boundary between gray matter (left) and white matter (right, dorsal column). Scale bar, 100 μm. Created with BioRender.com.

(**d**) Representative images of each cell type, classified by the following markers: OPCs as *Olig2*+ and *Pdgfra*+ cells; early (pre-myelinating) as *Olig2*+, *Pdgfra*- and low *Aspa*; mature oligodendrocytes as *Olig2*+, *Pdgfra*- and high *Aspa* cells; non-oligodendrocytes as *Olig2*- cells. Similar results were obtained within the same cell type for n = 14-30 cells quantified in (f). Scale bar, 10 μm.

(**e**) Heat map of fluorescence intensity from each probe in (c), where red is the highest intensity and white is the lowest within a probe, and each line corresponds to a single cell.

(**f**) Bar graph of mRNA fluorescence intensity of *Vamp2* and *Vamp3* in for each cell type. Data are presented as mean values +/- SEM from n = 23 (OPC, 30 (early OL), 22 (mature OL), and 14 (non-OL) cells. Each data point represents an individual cell.

(**g**) iBot;*Cnp*-Cre mouse and control (ctrl) littermate at P12 with ruler showing cm for scale.

(**h**) Body weight (mean ± SEM, n = 5) at postnatal day 10 for control littermates (5.79 ± 0.84 g) and iBot;*Cnp*-Cre mice (2.75 ± 0.15 g). Squares and circles denote males and females, respectively. Statistical measurement (p-value) was determined by an unpaired, two-tailed t-test.

(**i**) Survival curve of iBot;*Cnp*-Cre mice (n = 11) and control littermates (n = 21).

Supplementary Fig. 2

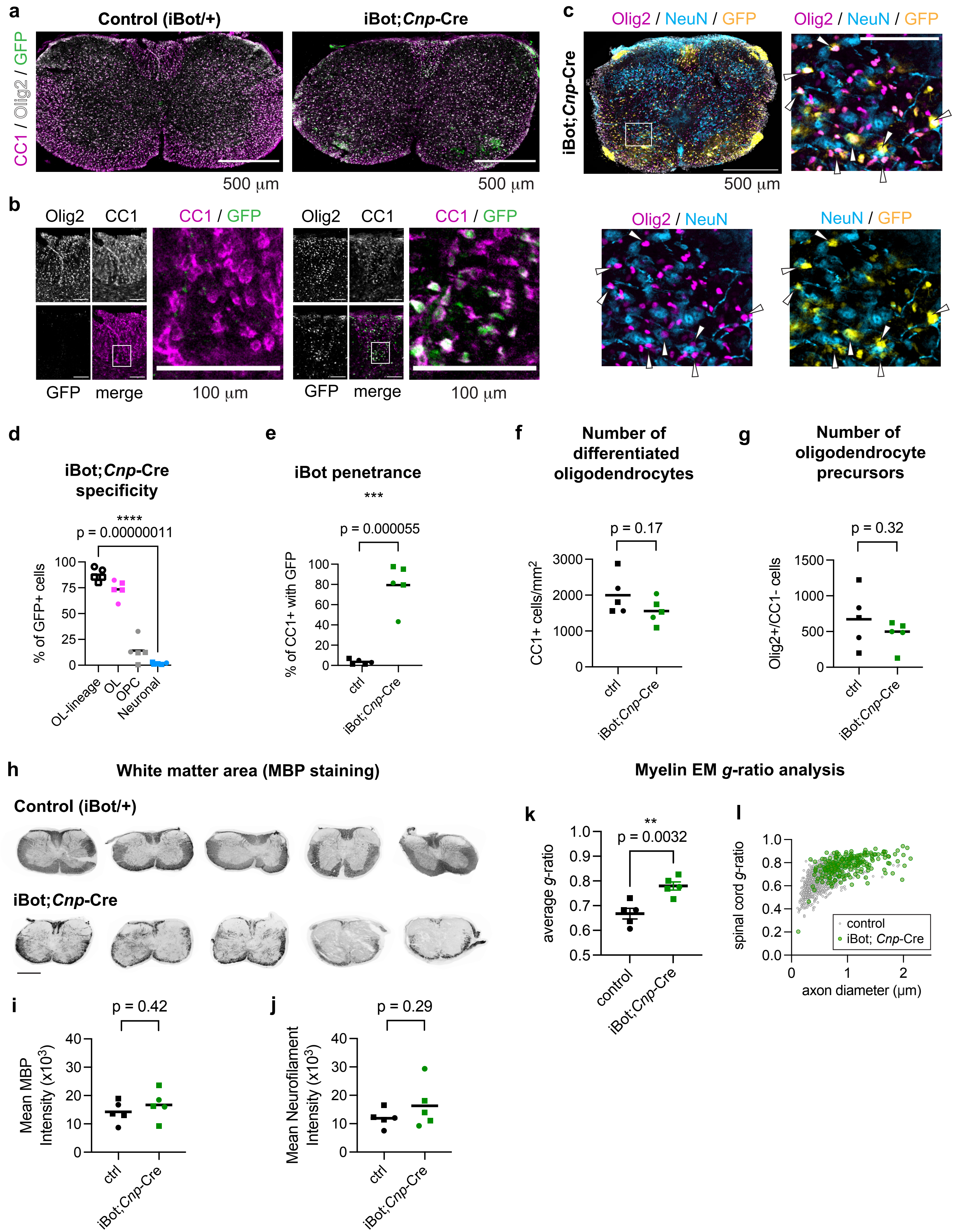

Supplementary Fig. 2 | Specificity and penetrance of botulinum toxin (iBot) expression in myelinating oligodendrocytes.

(**a-b**) Immunolabeling of P12 spinal cord cross sections from control (left) and iBot;*Cnp*-Cre (right) mice for CC1 (magenta), Olig2 (white), and GFP to mark iBot expression (green). Scale bar, 500 μm. (b) depicts the dorsal column with enlarged insets to show individual CC1+ cells. Scale bar, 100 μm. Similar results were obtained from n = 5 biological replicates quantified in (d) and shown in (h).

(**c**) Immunolabeling of P12 spinal cord cross sections from iBot;*Cnp*-Cre mice for Olig2 (magenta), NeuN (cyan), and GFP to mark iBot expression (yellow). Scale bar, 500 μm. Inset enlarges a region of the ventral horn, and white arrows point to Olig2+;GFP+ cells. Scale bar, 100 μm. Similar results were obtained from n = 5 biological replicates quantified in (d).

(**d**) Quantification of iBot;*Cnp*-Cre specificity by counting the % of total GFP+ overlapping with different cell markers (average ± SEM): Olig2 (oligodendrocyte-lineage, 87.8 ± 2.71%), Olig2 and CC1 (oligodendrocyte, 73.5 ± 4.00%), Olig2 without CC1 (OPC, 14.2 ± 5.20%), or NeuN (neuronal, 1.75 ± 0.435%). n = 5 biological replicates with 1067-2085 GFP+ cells analyzed in each. Statistical measurement (p-value) was determined by an unpaired, two-tailed t-test. Squares and circles denote males and females, respectively.

(**e**) Quantification of iBot penetrance by counting the % of total CC1+ overlapping with GFP (average ± SEM) in control (3.5 ± 1.1%) and iBot;*Cnp*-Cre (79.4 ± 9.7%) littermates for n = 5 biological replicates of each genotype with 1096-2881 CC1+ cells analyzed in each. Statistical measurement (p-value) was determined by an unpaired, two-tailed t-test. Squares and circles denote males and females, respectively.

(**f**) Quantification of the number of CC1+ cells per mm^2 (average ± SEM) for control (1999 ± 249) and iBot;*Cnp*-Cre (1558 ± 160) littermates with n = 5 biological replicates for each

genotype. Statistical measurement (p-value) was determined by an unpaired, two-tailed t-test. Square and circles denote males and females, respectively.

(**g**) Quantification of the number of CC1-/Olig2+ cells per mm^2 (average $\pm$ SEM) for control (672 $\pm$ 176) and iBot;*Cnp*-Cre (463 $\pm$ 87.4) littermates with n = 5 biological replicates for each genotype. Statistical measurement (p-value) was determined by an unpaired, two-tailed t-test. Squares and circles denote males and females, respectively.

(**h**) Biological replicates of MBP immunolabeling of P12 spinal cord cross sections from control (top row) and iBot;*Cnp*-Cre (bottom row) mice) for the representative image shown in Fig. 1c. Scale bar, 500 μm.

(**i**) Quantification of mean MBP intensity in arbitrary units (arb. units) over the entire spinal cord cross section (P12) shown in (h); average $\pm$ SEM for n = 5; control: (14.2 $\pm$ 1.75) x 10^3; iBot;*Cnp*-Cre: (16.7 $\pm$ 2.31) x 10^3). Statistical measurement (p-value) was determined by an unpaired, two-tailed t-test. Squares and circles denote males and females, respectively.

(**j**) Quantification of mean neurofilament 200 intensity (arb. units) over the entire spinal cord cross section (P12) shown in (h); average $\pm$ SEM for n = 5; control: (11.9 $\pm$ 1.42) x 10^3; iBot;*Cnp*-Cre: (16.3 $\pm$ 3.58) x 10^3). Statistical measurement (p-value) was determined by an unpaired, two-tailed t-test. Squares and circles denote males and females, respectively.

(**k**) Quantification of *g*-ratio for wrapped/wrapping axons in control vs. iBot;*Cnp*-Cre spinal cords at P12 as shown in Fig. 1f-g; average $\pm$ SEM for n = 5; control: 0.668 $\pm$ 0.0213; iBot;*Cnp*-Cre: 0.780 $\pm$ 0.0166). Statistical measurement (p-value) was determined by an unpaired, two-tailed t-test. Squares and circles denote males and females, respectively.

(**l**) Distribution of *g*-ratio vs. axon diameter for wrapped/wrapping axons in control vs. iBot;*Cnp*-Cre spinal cords at P12 as shown in Fig. 1f-g.

Supplementary Fig. 3

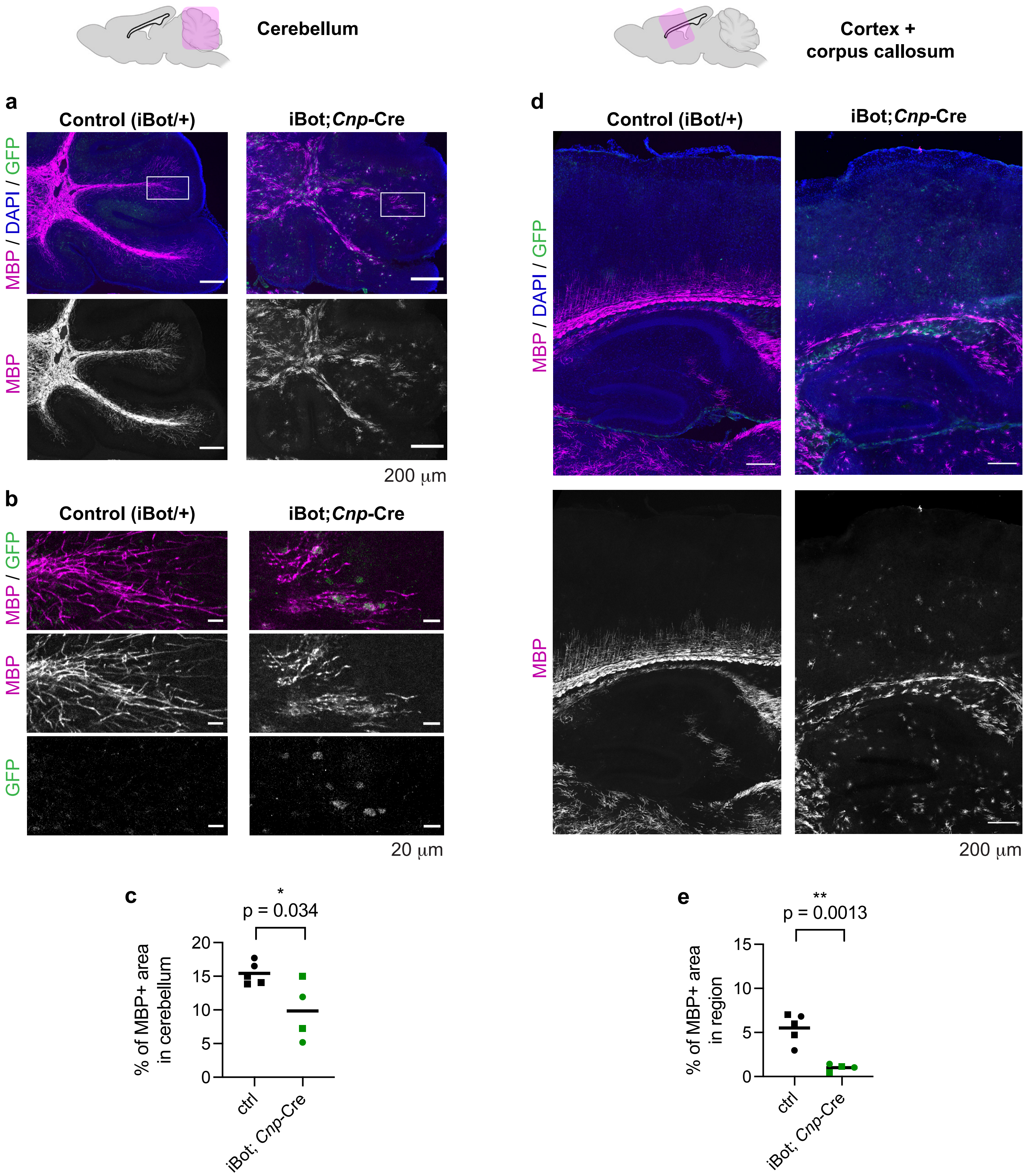

Supplementary Fig. 3 | VAMP2/3 is required for developmental myelination in the brain.

(**a**) Immunolabeling of a P12 cerebellum from control (left) and iBot;*Cnp*-Cre (right) mice for MBP (magenta), DAPI (blue), and GFP to mark iBot expression (green). Scale bar, 200 μm. Created with BioRender.com.

(**b**) Insets from (a) to show differential MBP morphology of iBot-expressing (GFP+) oligodendrocytes. Scale bar, 20 μm.

(**c**) Quantification of the percent of area immunolabeled by the myelin marker MBP (average ± SEM) across the cerebellum for control (15.4 ± 0.74%, n = 5) and iBot;*Cnp*-Cre (9.8 ± 2.2%, n = 4). Statistical measurement (p-value) was determined by an unpaired, two-tailed t-test. Squares and circles denote males and females, respectively.

(**d**) Immunolabeling of a sagittal slice of the P12 cortex from control (left) and iBot;*Cnp*-Cre (right) mice for MBP (magenta), DAPI (blue), and GFP to mark iBot expression (green). Scale bar, 200 μm. Created with BioRender.com.

(**e**) Quantification of the percent of area immunolabeled by the myelin marker MBP (average ± SEM) across cortical sections for control (5.5 ± 0.75%, n = 5) and iBot;*Cnp*-Cre (1.0 ± 0.2%, n = 4). Statistical measurement (p-value) was determined by an unpaired, two-tailed t-test. Squares and circles denote males and females, respectively.

Supplementary Fig. 4

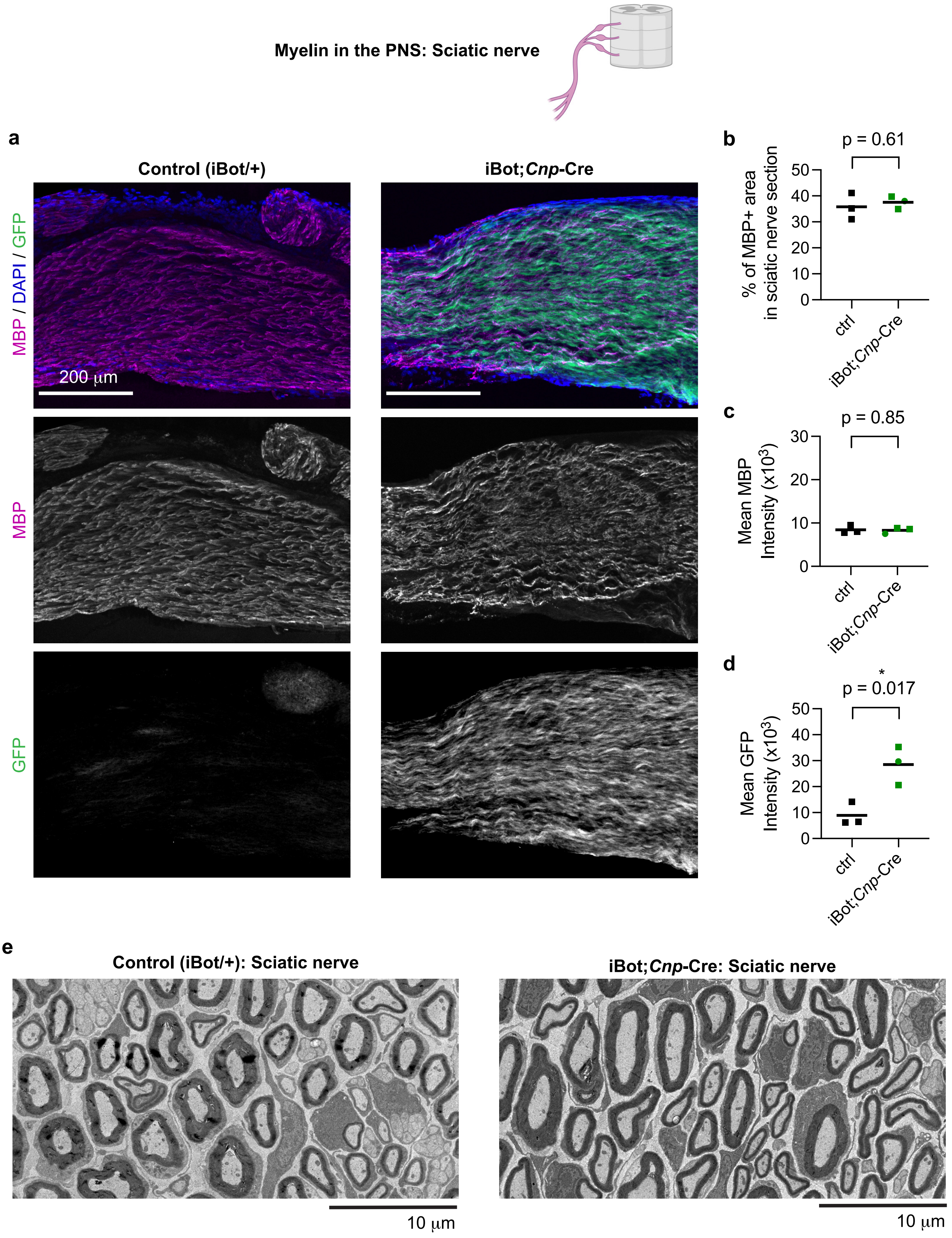

Supplementary Fig. 4 | Inactivation of VAMP2/3 has no gross effect on PNS myelination.

(**a**) Immunolabeling of a P12 sciatic nerve longitudinal section from control (left) and iBot;*Cnp*-Cre (right) mice for MBP (magenta), DAPI (blue), and GFP to mark iBot expression (green). Scale bar, 200 μm. Created with BioRender.com.

(**b**) Quantification of the percent of area immunolabeled by the myelin marker MBP (average ± SEM) across the sciatic nerve for control (35.8 ± 2.9%, n = 3) and iBot;*Cnp*-Cre (37.6 ± 1.4%, n = 3). Statistical measurement (p-value) was determined by an unpaired, two-tailed t-test. Squares and circles denote males and females, respectively. Squares and circles denote males and females, respectively.

(**c**) Quantification of mean intensity of MBP (average ± SEM, arb. units = arbitrary units) in sciatic nerves for control (8442 ± 550 arb. units, n = 3) and iBot;*Cnp*-Cre (8311 ± 407 arb. units, n = 3). Statistical measurement (p-value) was determined by an unpaired, two-tailed t-test. Square and circles denote males and females, respectively.

(**d**) Quantification of mean intensity of GFP (average ± SEM, arb. units = arbitrary units) in sciatic nerves for control (8942 ± 2599, n = 3) and iBot;*Cnp*-Cre (28534 ± 4244, n = 3). Statistical measurement (p-value) was determined by an unpaired, two-tailed t-test. Square and circles denote males and females, respectively.

(**e**) Transmission electron microscopy images from P12 mouse sciatic nerve cross sections (left: control; right: iBot;*Cnp*-Cre). Scale bar, 10 μm. Similar results were observed for n = 2 biological replicate pairs.

Supplementary Fig. 5

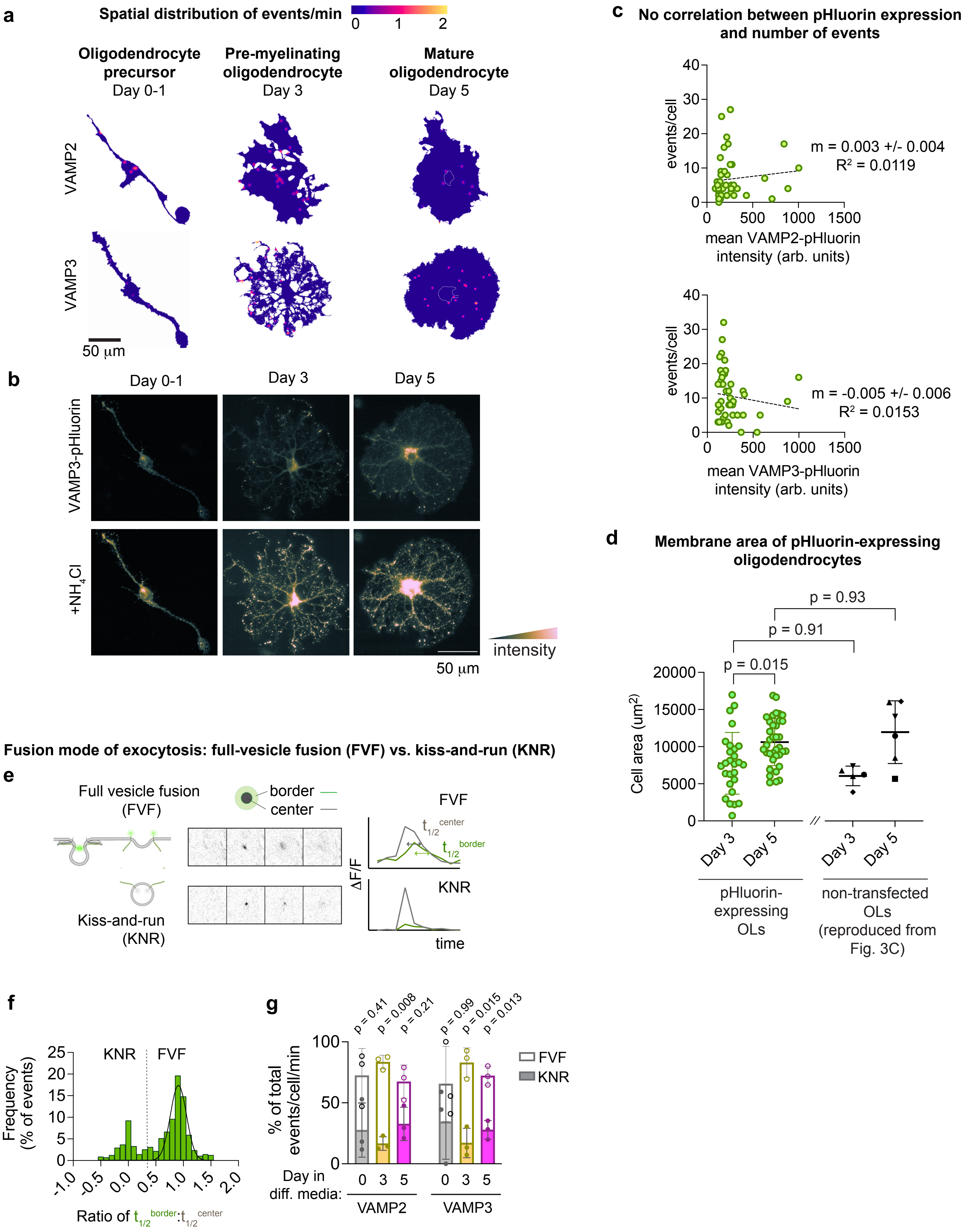

Supplementary Fig. 5 | Spatial distribution and fusion modes of VAMP2/3-vesicles in primary oligodendrocytes.

(**a**) Heat map of the density of exocytotic events in primary oligodendrocytes differentiated for 0-1, 3, or 5 days before imaging.

(**b**) Images of primary oligodendrocytes expressing VAMP3-pHluorin (top) followed by a 10-second treatment with 30 mM NH_4Cl (bottom) to de-quench all pHluorin-associated vesicles. Similar results were observed for n = 3 biological replicates each with 26-33 cells.

(**c**) Plot of the number of exocytotic events vs. the mean background pHluorin intensity of each cell at t = 0 as a proxy of expression level for VAMP2-pHluorin (top) and VAMP3-pHluorin (bottom). Higher number of exocytotic events did not correlate with higher VAMP-pHluorin expression levels.

(**d**) Comparison of membrane area between pHluorin-expressing (green, pooled data from VAMP2 and VAMP3) and non-transfected primary oligodendrocytes (black) at Day 3 and Day 5 of differentiation in culture. Data shown for non-transfected primary oligodendrocytes were reproduced from the membrane area averages for control oligodendrocytes in Fig. 3c. Mean cell area $\pm$ SEM: at Day 3, pHluorin-expressing 7,761 + 798 μm^2 (n = 27), non-transfected control 6,099 + 595 μm^2; at Day 5, pHluorin-expressing 10,607 + 528 μm^2 (n = 36), non-transfected control 12,022 + 1,731 μm^2. Statistical significance was determined by a one-way ANOVA with Tukey's correction for multiple comparisons.

(**e**) Examples of exocytotic events resulting in different fusion modes of exocytosis. On the top, a full-vesicle fusion (FVF) event exhibits radial spreading of fluorescence from the initial fluorescent punctum. On the bottom, a kiss-and-run (KNR) event appears and disappears without fluorescence spreading. The half-life of the event center ($t_{1/2}^{center}$) measures the duration of initial fluorescent punctum, and the half-life of the bordering event ($t_{1/2}^{border}$) measures the decay of the surrounding membrane fluorescence. In FVF events, the $t_{1/2}^{border}$ (green trace)

should be similar to the $t_{1/2}^{center}$ (gray trace), yielding a ratio of half-lives of ~ 1. Created with BioRender.com.

(**f**) Frequency distribution of the ratio of half-lives from the bordering event to the center event ($t_{1/2}^{center}$: $t_{1/2}^{border}$) as a measure of radial fluorescence spreading. Ratios of all pHluorin events (n = 396 for VAMP2 and 539 for VAMP3) distributed into two clusters. FVF was assigned to the cluster ~ 1 (mean $\pm$ SD of Gaussian fit for VAMP2: 0.89 $\pm$ 0.14; VAMP3: 0.92 $\pm$ 0.15). Ratios below three standard deviations of the FVF mean (marked by the dotted line) were classified as KNR.

(**g**) Stacked bar graph for composition of events classified as FVF (top open bars and circles) or KNR (bottom filled bars and circles) at Day 0, 3, and 5 of differentiation in culture corresponding to OPC, pre-myelinating, and mature stages, respectively. Statistical significance was determined by a two-way ANOVA between KNR and FVF at each day of differentiation with Bonferroni's correction for multiple comparisons. For statistically significant differences, the mean $\pm$ SEM% of FVF events was 83.4 $\pm$ 4.5% for VAMP2 at Day 3, 82.9 $\pm$ 9.8% for VAMP3 at Day 3, and 72.1 $\pm$ 6.3% for VAMP3 at Day 5 from n = 3 biological replicates.

Supplementary Fig. 6

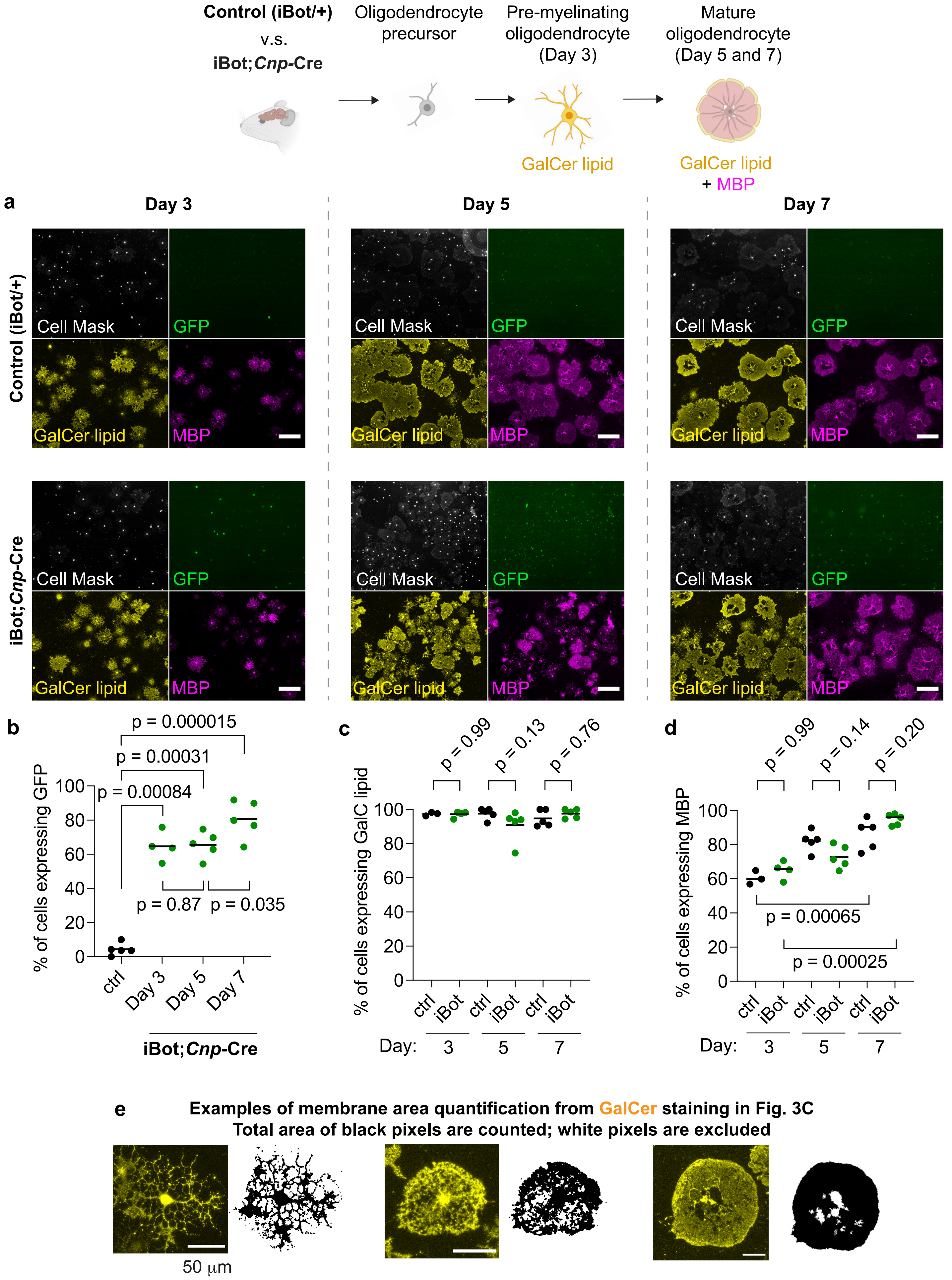

Supplementary Fig. 6 | VAMP2/3 inactivation in pre-myelinating oligodendrocytes does not affect oligodendrocyte differentiation in culture.

(**a**) OPCs were purified from the brains of control or iBot;*Cnp*-Cre mice and differentiated in culture for 3, 5, or 7 days. Cells were fixed and stained for Cell Mask Blue (a marker of morphology), GalCer lipid (a marker of pre-myelinating oligodendrocytes), and MBP (a marker of mature oligodendrocytes). Scale bar, 200 μm. Created with BioRender.com.

(**b**) Quantification of iBot expression as % of cells expressing GFP (mean ± SEM) for n = 5 biological replicates in each condition. Control: 4.3 ± 1.6%. iBot;*Cnp*-Cre Day 3: 64.7 ± 4.3%, Day 5: 65.6 ± 3.4%, Day 7: 80.6 ± 4.9%. Statistical significance was determined by a one-way ANOVA with Bonferroni's correction for multiple comparisons.

(**c**) Quantification of pre-myelinating oligodendrocytes as % of cells expressing GalCer lipid (mean ± SEM) for n = 5 biological replicates in each condition. Control Day 3: 97.4 ± 0.6%, Day 5: 97.6 ± 1.5%, and Day 7: 94.8 ± 2.2%. iBot;*Cnp*-Cre Day 3: 97.2 ± 0.9%, Day 5: 90.9 ± 4.2%, Day 7: 97.7 ± 1.2%. Statistical significance was determined by a one-way ANOVA with Bonferroni's correction for multiple comparisons.

(**d**) Quantification of mature oligodendrocytes as % of cells expressing MBP (mean ± SEM) for n = 5 biological replicates in each condition. Control Day 3: 60.6 ± 2.3%, Day 5: 82.1 ± 2.7%, and Day 7: 86.2 ± 4.0%. iBot;*Cnp*-Cre Day 3: 65.1 ± 2.6%, Day 5: 72.9 ± 3.0%, Day 7: 94.7 ± 1.5%. Statistical significance was determined by a one-way ANOVA with Bonferroni's correction for multiple comparisons.

(**e**) Examples of membrane area quantification from GalCer staining in Fig. 3C to show how unstained regions arising from the reticulated membrane network are excluded from area quantification.

Supplementary Fig. 7

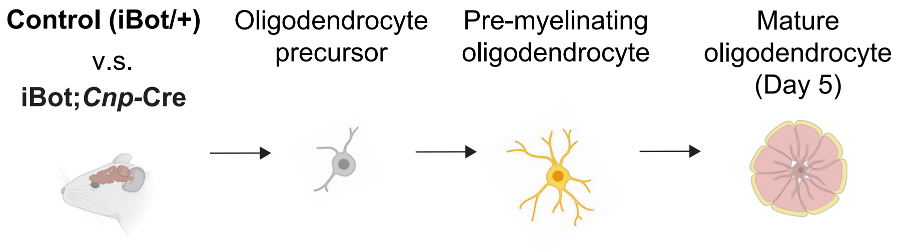

a

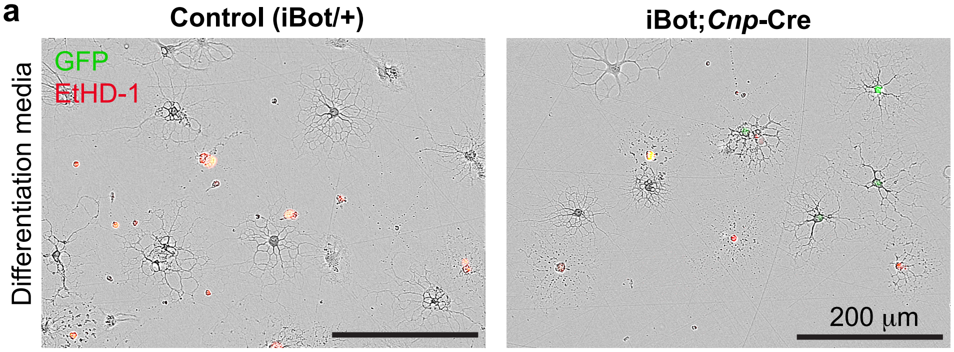

b

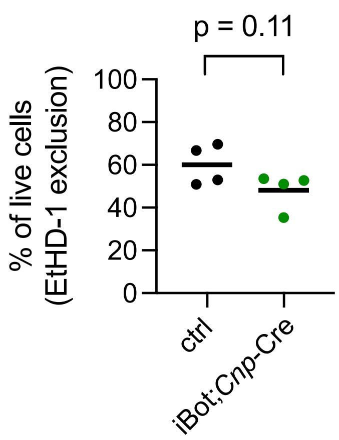

c

Genotype	Rep	Total cells	EtHD-1+	%EtHD-1+	%live
ctrl	1	194	59	30%	70%
	2	171	57	33%	67%
	3	204	96	47%	53%
	4	165	81	49%	51%
iBot	1	153	71	46%	54%
	2	186	91	49%	51%
	3	245	116	47%	53%
	4	195	126	65%	35%

d

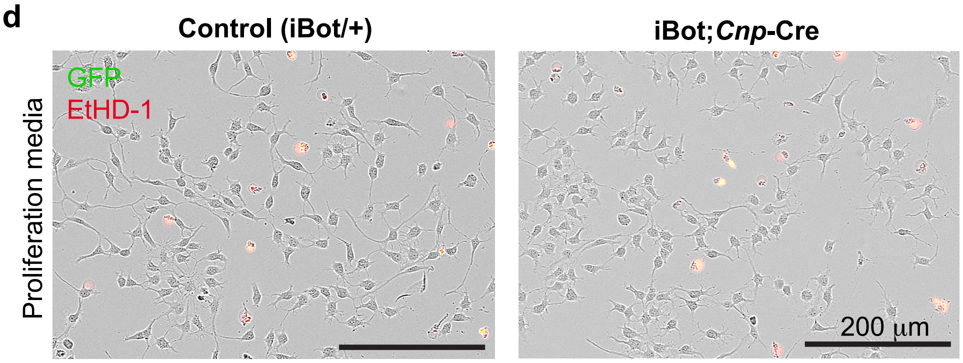

e

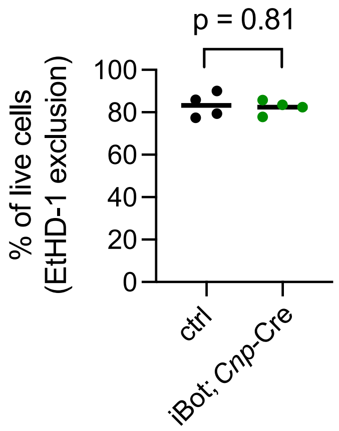

f

Genotype	Rep	Total cells	EtHD-1+	%EtHD-1+	%live
ctrl	1	1597	159	10%	90%
ctrl	2	1002	226	23%	77%
ctrl	3	1637	230	14%	86%
ctrl	4	813	168	21%	79%
iBot	1	1177	167	14%	86%
iBot	2	940	155	16%	84%
iBot	3	1450	255	18%	82%
iBot	4	675	150	22%	78%

Supplementary Fig. 7 | VAMP2/3 inactivation does not affect oligodendrocyte cell survival.

(**a**) Dead cell staining of primary oligodendrocytes at Day 5 in differentiation media with ethidium homodimer (EtHD-1, red). Scale bars, 200 μm. Created with BioRender.com.

(**b**) Quantification of live oligodendrocytes as % of cells that excluded ethidium homodimer (mean ± SEM) with n = 4 biological replicates for control (60.0 ± 4.7%) and iBot;*Cnp*-Cre (48.2 ± 4.3%). Statistical measurement (p-value) was determined by an unpaired, two-tailed t-test.

(**c**) Number of oligodendrocytes quantified in each biological replicate.

(**d**) Dead cell staining of oligodendrocyte precursors in proliferation media with ethidium homodimer (EtHD-1, red). Scale bars, 200 μm.

(**e**) Quantification of live precursors as % of cells that excluded ethidium homodimer (mean ± SEM) with n = 4 biological replicates for control (83.2 ± 2.9%) and iBot;*Cnp*-Cre (82.4 ± 1.7%). Statistical measurement (p-value) was determined by an unpaired, two-tailed t-test.

(**f**) Number of oligodendrocyte precursors quantified in each biological replicate.

Supplementary Fig. 8

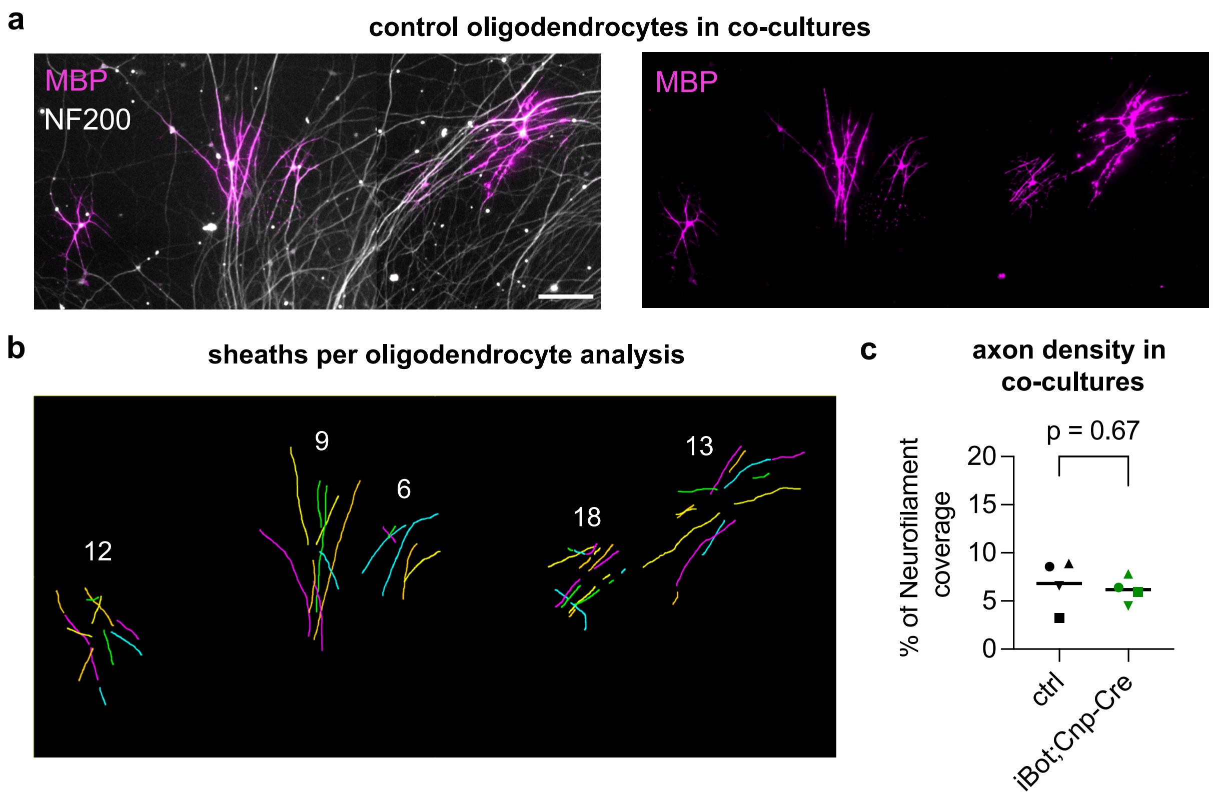

Supplementary Fig. 8 | Example of sheath number per cell analysis

(**a**) Primary oligodendrocytes purified from control mouse brain cultured on CNS-derived axons (retinal ganglion cell re-aggregates) for 7 days and stained for MBP (magenta) and NF200 (white). Scale bar 100 μm. Image is representative of n = 4 biological replicates.

(**b**) Examples of sheath analysis for individual oligodendrocytes in co-cultures, where each color depicts a distinct sheath. The white number corresponds to the number of sheaths counted per cell.

(**c**) Quantification of axon density by neurofilament-200 staining in co-cultures shown in Fig. 4b-d, with different shape symbols corresponding to biologically independent replicates. Statistical measurement (p-value) was determined by an unpaired, two-tailed t-test.

Supplementary Fig. 9

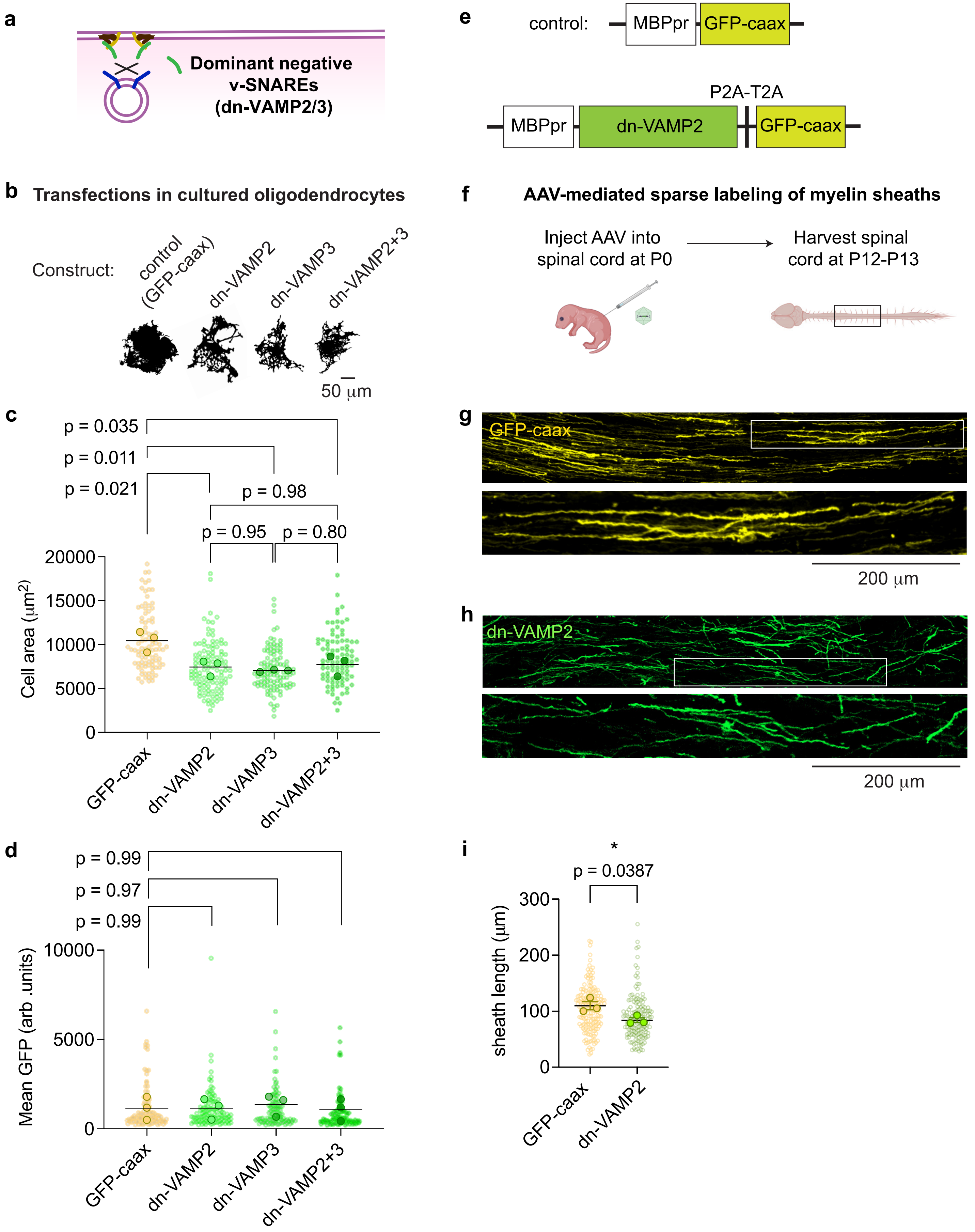

Supplementary Fig. 9 | Expression of dominant negative VAMP (dn-VAMP2/3) as an orthogonal method of inhibiting VAMP2/3-mediated exocytosis

(**a**) Schematic of dominant negative v-SNAREs (green, dn-VAMP2/3) inhibiting membrane fusion by sequestering binding sites on the target membrane from endogenous v-SNAREs (blue). Created with BioRender.com.

(**b**) GalCer lipid-stained masks of primary rat oligodendrocytes transfected with MBP promoter-driven dn-VAMP2, dn-VAMP3, or both dn-VAMP2 and 3 (dn-VAMP2+3), and differentiated for 5 days in culture to show cell morphologies. Scale bar, 50 μm.

(**c**) Quantification of membrane surface area marked by GalCer lipid, where each light-shaded point corresponds to a single cell, each dark-shaded point represents the mean area of cells from one biological replicate, and the line represents the mean area from n = 3 biological replicates for each condition. Mean cell area $\pm$ SEM after Day 5 of differentiation: GFP-caax (control) 10,457 $\pm$ 690 μm^2; dn-VAMP2 7,454 $\pm$ 531 μm^2; dn-VAMP3 7,040 $\pm$ 78.5 μm^2; dn-VAMP2+3 7,745 $\pm$ 687 μm^2. Statistical significance was determined by a one-way ANOVA with Tukey's correction for multiple comparisons.

(**d**) Quantification of mean GFP to compare expression levels of dn-VAMP constructs,where each light-shaded point corresponds to a single cell, each dark-shaded point represents the mean area of cells from one biological replicate, and the line represents the mean area from n = 3 biological replicates for each condition. Mean GFP intensity $\pm$ SEM after Day 5 of differentiation (arb. units): GFP-caax (control) 1,153 $\pm$ 371; dn-VAMP2 1,154 $\pm$ 338; dn-VAMP3 1,357 $\pm$ 347; dn-VAMP2+3 1,093 $\pm$ 356. Statistical significance was determined by a one-way ANOVA with Tukey's correction for multiple comparisons.

(**e**) Schematic of constructs used for AAV-mediated sparse labeling of oligodendrocytes, where the control corresponds to MBP promoter (MBPpr)-driven GFP-caax (top), and the experimental

condition uses MBPpr-driven dn-VAMP2 followed by a tandem self-cleaving peptide (P2A-T2A) and GFP-caax.

(**f**) Schematic of neonatal mouse pup spinal cord injections for AAV-mediated sparse labeling of oligodendrocytes, where the lumbar spinal cord is injected at P0 and the thoracic spinal cord region is harvested at P12-P13 for sheath analysis. Created with BioRender.com.

(**g**) Representative image of oligodendrocytes expressing GFP-caax in the P12 mouse spinal cord. Inset depicts a single, discrete oligodendrocyte and its corresponding sheaths. Scale bar for inset, 200 μm. Similar results were obtained for n = 3 biological replicates quantified in (i).

(**h**) Representative image of oligodendrocytes expressing dn-VAMP2-P2A-T2A-GFP-caax in the P12 mouse spinal cord. Inset depicts a single, discrete oligodendrocyte and its corresponding sheaths. Scale bar for inset, 200 μm. Similar results were obtained for n = 3 biological replicates quantified in (i).

(**i**) Quantification of sheath lengths labeled by GFP-caax for control vs. dn-VAMP2, where each light-shaded point corresponds to a single cell, each dark-shaded point represents the mean area of cells from one biological replicate, and the line represents the mean area from n = 3 biological replicates for each condition. Mean sheath length ± SEM: GFP-caax (control) 110 ± 7.31 μm; dn-VAMP2 84.0 ± 4.45 μm. Statistical measurement (p-value) was determined by an unpaired, two-tailed t-test.

Supplementary Fig. 10

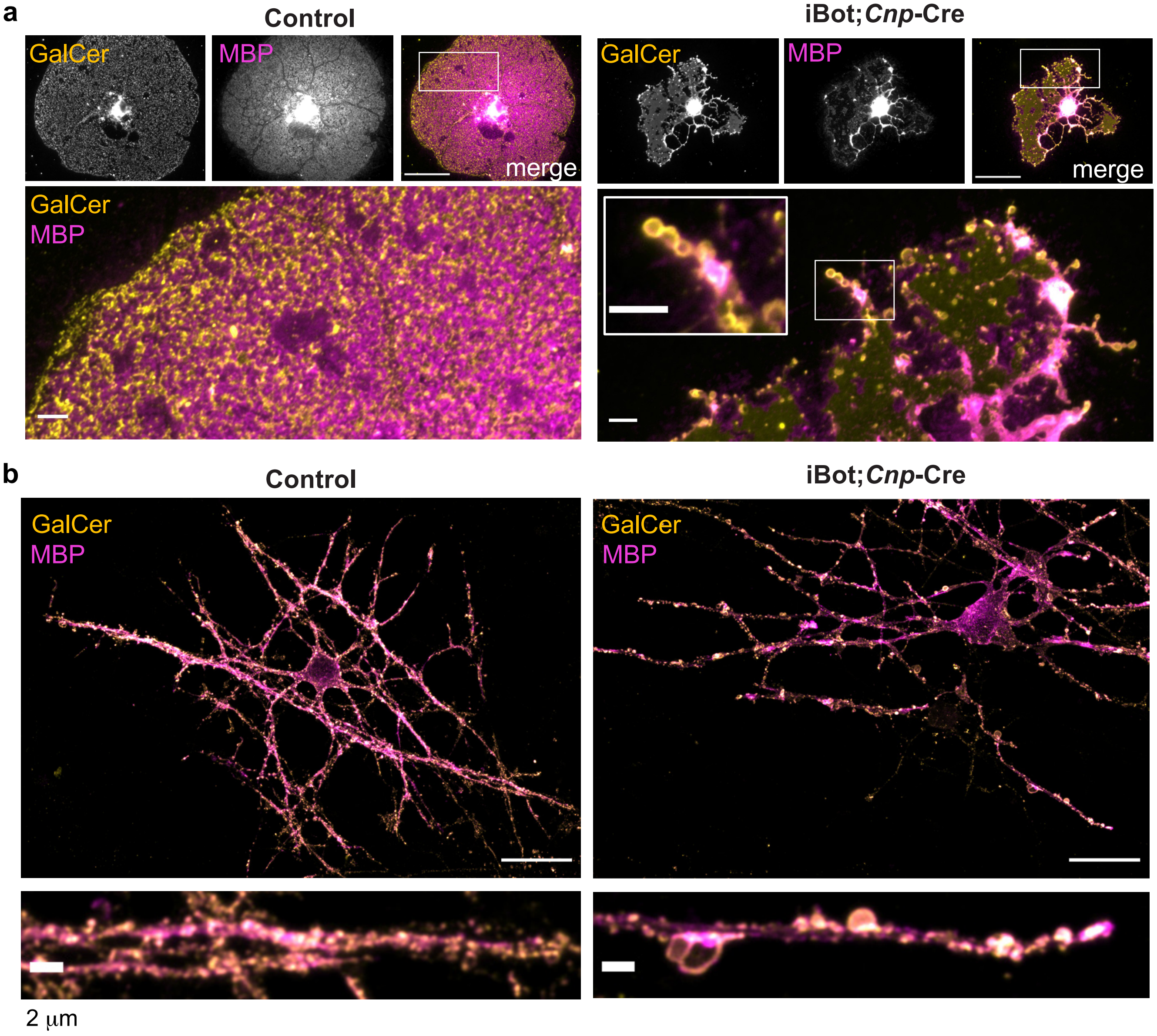

Supplementary Fig. 10 | VAMP2/3 inactivation induces vesicle accumulation in oligodendrocyte processes and sheaths in culture.

(**a**) Confocal images of mature oligodendrocytes from control (left) and iBot;*Cnp*-Cre (right) mice stained for GalCer lipid and MBP. Images are representative of cells observed from n = 5 biological replicates. Scale bars: top row, 50 μm; bottom row and inset, 5 μm.

(**b**) Confocal images of oligodendrocyte-neuron co-cultures from control (left) and iBot;*Cnp*-Cre (right) oligodendrocytes stained for GalCer lipid and MBP. Images are representative of cells observed from n = 4 biological replicates. Scale bars: top row, 20 μm; bottom row and inset, 2 μm.

Supplementary Fig. 11

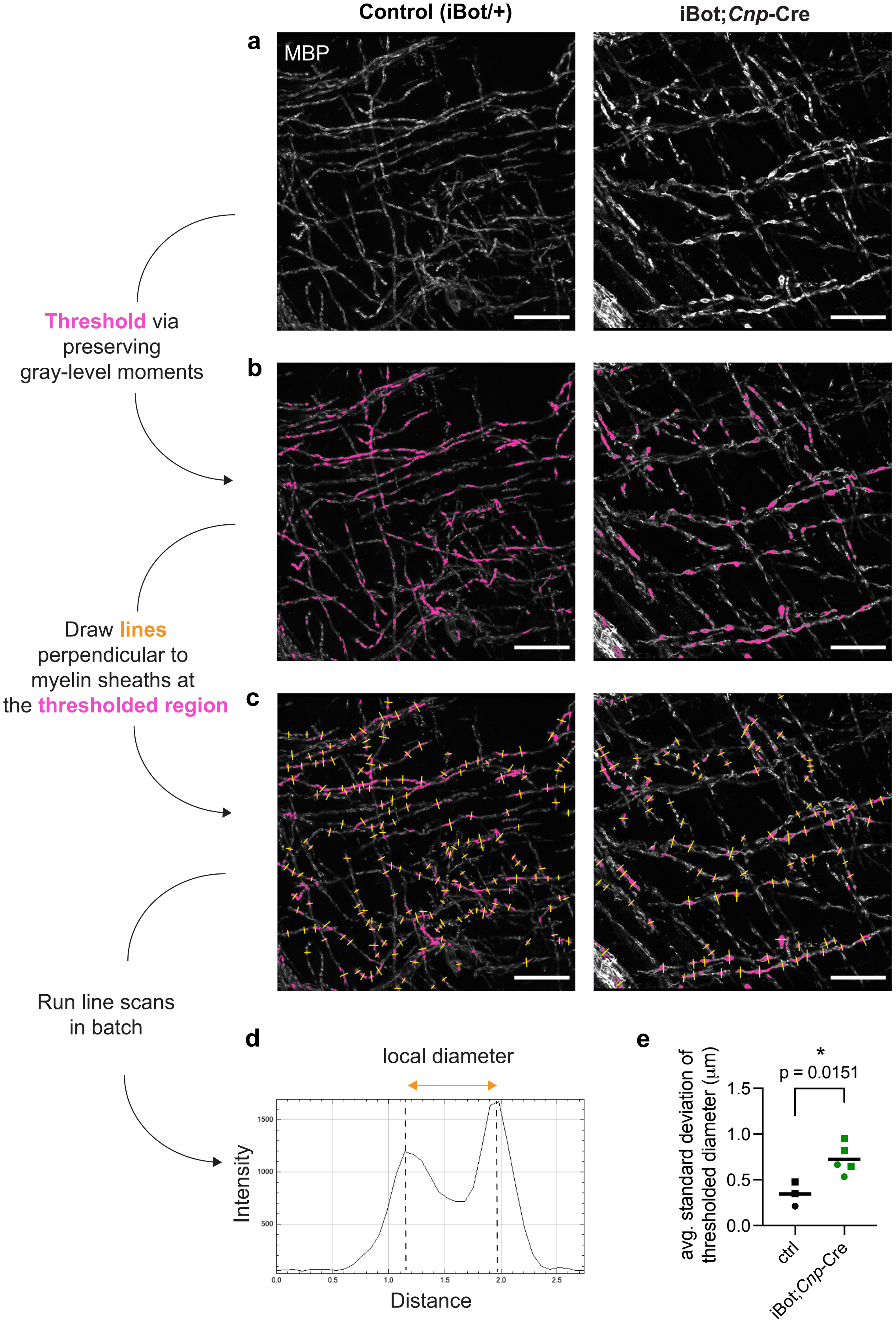

Supplementary Fig. 11 | Methodology for quantifying local diameter of myelin sheaths in Fig. 5c.

(**a**) Airyscan confocal images of P12 mouse cingulate cortex from control (left) and iBot;*Cnp*-Cre (right) littermates immunostained for MBP. Images are representative of n = 3 control and n = 5 iBot;*Cnp*-Cre biological replicates. Scale bar, 20 μm.

(**b**) The pink patches highlight regions thresholded for MBP intensity by preserving gray-level moments (Fiji).

(**c**) Yellow depicts lines that are drawn perpendicular to the longest length of each thresholded pink region. Each yellow line is perpendicular to a myelin sheath.

(**d**) A line scan of each yellow line traces the intensity vs. distance along the line, resulting in two local maxima that correspond to the paired parallel MBP tracks within a sheath. The distance between the two local maxima corresponds to the local diameter of the myelin sheath.

(**e**) The average of the standard deviation (st. dev.) among local diameters measured within a biological replicate. Squares and circles denote males and females, respectively. Mean st. dev. ± SEM: control 0.345 ± 0.0769 μm from n = 3 biological replicates; iBot;*Cnp*-Cre 0.723 ± 0.0730 μm from n = 5 biological replicates. Statistical measurement (p-value) was determined by an unpaired, two-tailed t-test.

Supplementary Fig. 12

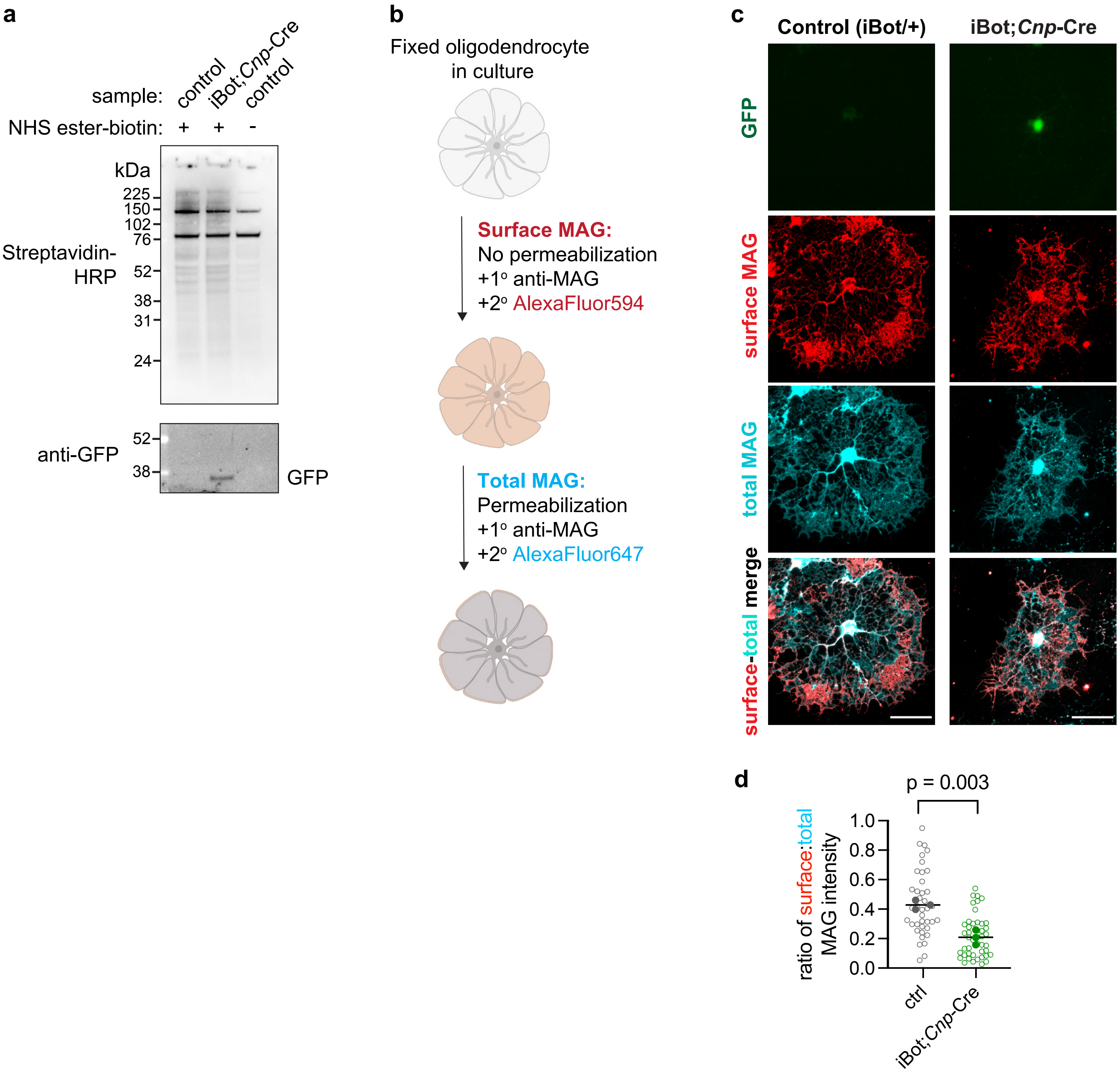

Supplementary Fig. 12 | Mass spectrometry of surface-biotinylated proteins from oligodendrocytes reveals that the transport of myelin-associated glycoprotein depends on VAMP2/3.

(**a**) Western blot of mass spectrometry inputs probed for biotinylated substrates (with streptavidin-HRP) and for iBot expression via anti-GFP from one experiment. kDa = kilodalton.

(**b**) Schematic for co-staining cell surface and intracellular myelin associated glycoprotein (MAG), where permeabilization refers to treatment with 0.1% Triton X-100.

(**c**) Representative images of surface and intracellular staining for MAG in control (left) and iBot;*Cnp*-Cre (right) oligodendrocytes. In control cells, surface MAG localizes to the cell periphery, outlining the outer rim of the cell.

(**d**) Quantification of surface MAG intensity relative to total MAG intensity (mean $\pm$ SEM) for control (0.43 $\pm$ 0.02) and iBot;*Cnp*-Cre (0.21 $\pm$ 0.03) from n = 3 biological replicates. Statistical measurement (p-value) was determined by an unpaired, two-tailed t-test.

Supplementary Fig. 13

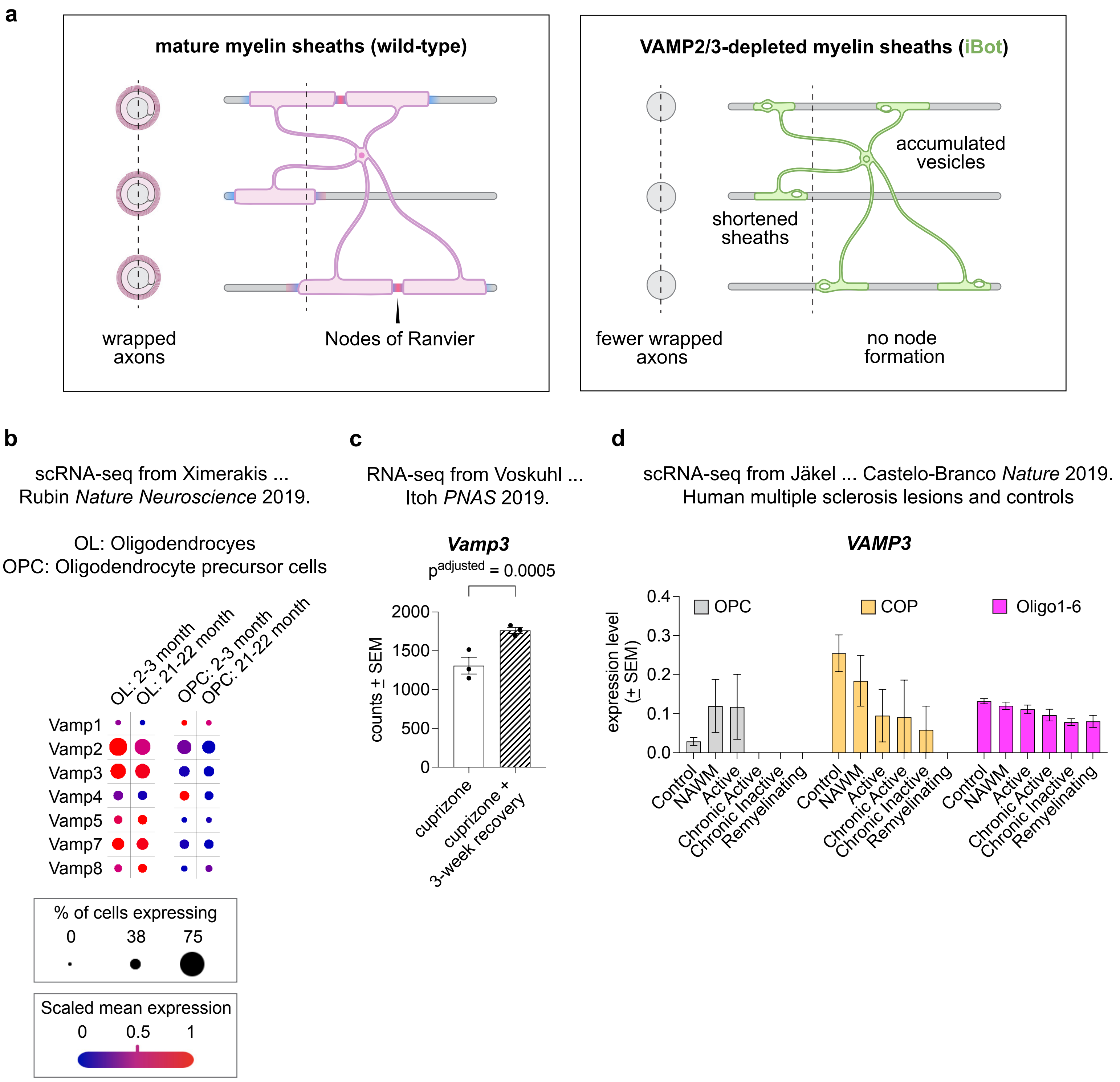

Supplementary Fig. 13 | VAMP2/3 expression from published datasets of transcriptomic changes in aging and multiple sclerosis.

(**a**) Depletion of VAMP2/3 by botulinum toxin expression (iBot) causes an accumulation of unfused vesicles in myelin sheaths, preventing sheath elongation and node of Ranvier formation. Shorter sheaths result in reduced myelin coverage of axons, which appears as a higher percentage of unmyelinated axons by TEM. See Methods for a quantitative estimation of relative myelin coverage by control and iBot;*Cnp*-Cre. Created with BioRender.com.

(**b**) Dot plot of mean expression of v-SNARE isoforms in oligodendrocytes and OPCs from single-cell RNAseq of adult (2-3 month) and aged (21-22 month) mice published in Ximerakis *et al. Nat. Neuroscien*ce 2019[3]. Plots were generated from the interactive viewer on https://singlecell.broadinstitute.org/single_cell/study/SCP263/aging-mouse-brain.

(**c**) Replotted RNA-seq counts from Voskuhl *et al. PNAS* 2019[4] for *Vamp3* mRNA associated with oligodendrocyte-derived ribosomes. Samples were isolated from the corpus callosum of mice undergoing demyelination (cuprizone) versus remyelination (cuprizone + 3-week recovery), where $p^{adjusted}$ reports the p-value corrected for false discovery rate.

(**d**) Replotted single cell RNA-seq (scRNA-seq) data from Jäkel *et al*. *Nature* 2019[5] for *VAMP3* mRNA in white matter from post-mortem tissue of human controls (control) versus patients with progressive multiple sclerosis (MS). The following white matter areas were from MS patients: normal-appearing white matter (NAWM), active, chronic active, chronic inactive, and remyelinating. The different cell types depicted are oligodendrocyte precursors (OPC), differentiation-committed oligodendrocytes (COP), and differentiated oligodendrocytes (Oligo1-6).

vSNARE	VAMP2-pHluorin						VAMP3-pHluorin					
Differentiation stage	OPC		pre-myelinating		mature		OPC		pre-myelinating		mature	
Subcellular location	soma	process	soma	process	soma	sheet	soma	process	soma	process	soma	sheet
Mean (events/cell/min)	1.62	3.62	1.08	6.53	0.712	7.82	1.58	5.21	0.886	9.49	0.844	11.6
Std. Error of Mean	0.347	0.737	0.424	2.1	0.118	1.34	0.248	0.644	0.414	0.721	0.228	2.1
Lower 95% CI	0.129	0.452	-0.741	-2.5	0.203	2.05	0.516	2.44	-0.896	6.39	-0.137	2.6
Upper 95% CI	3.12	6.79	2.9	15.6	1.22	13.6	2.65	7.98	2.67	12.6	1.82	20.7
# of cells (n)	9		27		22		13		22		34	

Supplementary Table 1: Descriptive statistics for exocytotic events in cultured oligodendrocytes (CI = confidence interval)

vSNARE	VAMP2-pHluorin						VAMP3-pHluorin					
Differentiation	OPC/pre-myelinating		early OL		OL		OPC/pre-myelinating		early OL		OL	
Subcellular location	soma	process	soma	sheath	soma	sheath	soma	process	soma	sheath	soma	sheath
Mean (events/cell/min)	0.0396	0.621	0.0461	0.393	0.0592	0.162	0.0943	1.27	0.193	0.649	0.159	0.364
Std. Error of Mean	0.0254	0.249	0.0358	0.137	0.0289	0.0426	0.0363	0.135	0.081	0.306	0.101	0.0567
Lower 95% CI	-0.0412	-0.172	-0.0533	0.0126	-0.00917	0.0614	0.0107	0.955	-0.836	-3.24	-0.0886	0.226
Upper 95% CI	0.12	1.41	0.145	0.773	0.128	0.263	0.178	1.58	1.22	4.53	0.407	0.503
# of cells (n)	4		5		8		9		2		7	

Supplementary Table 2: Descriptive statistics for exocytotic events in zebrafish spinal cords (CI = confidence interval)

	Vamp2		Vamp3	
**Total events within myelin sheaths**	31		45	
**# of cells**	10 cells (in 8 animals)		8 (in 7 animals)	
**# of sheaths**	25 (out of 261)		25 (out of 158)	
**summed length of sheaths (μm)**	421.2		597.8	
**summed length of paranodes (μm)**	143.6		146.5	
**summed length of internodes (μm)**	277.6		451.3	
	**paranode**	**internode**	**paranode**	**internode**
**%length (predicted frequency)**	34.1	65.9	24.5	75.5
**%events (observed frequency, mean ± SEM)**	50 ± 22.4	50 ± 22.4	24.9 ± 12.7	75.1 ± 12.7

Supplementary Table 3: Spatial frequency of exocytosis in myelin sheaths

Day in differentiation media	3		5		7	
genotype	**ctrl**	**iBot**	**ctrl**	**iBot**	**ctrl**	**iBot**
Mean surface area (μm^2)	6099	5047	12022	3557	16261	8735
Std. Error of Mean	595	240	1731	325	849	1144
Lower 95% CI	4448	4380	7572	2656	13904	5560
Upper 95% CI	7750	5714	16471	4458	18618	11911
total # of cells from n = 5 biological replicates	310	256	344	254	282	252

Supplementary Table 4: Descriptive statistics for membrane surface area of primary oligodendrocytes in culture (CI = confidence interval)

Gene	Description	control biotinyl - rep1	control biotinyl - rep2	iBot biotinyl - rep1	iBot biotinyl - rep2	iBot biotinyl - rep3	non-biotinyl - rep1	non-biotinyl - rep2	non-biotinyl - rep3	log2(fold enrichment)	q-value (adjusted p-value)
Immt	MIC complex subunit Mic60	59	19	19	7	NaN	NaN	NaN	NaN	-6.25	0.050
Nfasc	Neurofascin	27	10	NA	NaN	NaN	NaN	NaN	NaN	-4.90	0.049
Cntn1	Contactin-1	51	18	7	3	NaN	NaN	NaN	NaN	-4.43	0.052
Atp5f1b	ATP synthase subunit beta, mitochondrial	124	36	56	16	24	NaN	NaN	1	-4.40	0.048
Mbp	Isoform 4 of Myelin basic protein	77	48	21	4	3	6	7	15	-4.05	0.048
Ank3 (AnkG)	Ankyrin-3 (also Ankyrin G)	12	9	NA	2	NaN	NaN	2	3	-3.82	0.053
Hspa2	Heat shock-related 70 kDa protein 2	9	9	NA	3	1	NaN	3	5	-3.25	0.046
Rtn4	Reticulon-4	88	62	9	6	3	5	17	12	-3.18	0.052
Tppp	Tubulin polymerization-promoting protein	10	8	4	1	1	NaN	3	NaN	-3.01	0.046
Mag	Myelin-associated glycoprotein	13	20	6	2	1	NaN	2	2	-2.98	0.049
Ndufa10	NADH dehydrogenase 1 alpha subcomplex subunit 10, mitochondrial	5	6	NA	NaN	NaN	NaN	NaN	NaN	-2.97	0.055
Bin1	Myc box-dependent-interacting protein 1	7	10	2	NaN	1	NaN	NaN	NaN	-2.90	0.048
Sept8	Septin-8	10	6	NA	2	2	NaN	NaN	4	-2.81	0.045
Dst	Dystonin	52	52	NA	NaN	NaN	NaN	NaN	16	-4.70	0.054
Tns3	Tensin-3	14	28	NA	1	NaN	NaN	NaN	7	-3.91	0.048
Frmd4a	FERM domain-containing protein 4A	13	7	NA	NaN	NaN	NaN	3	NaN	-3.82	0.054
Capzb	F-actin-capping protein subunit beta	6	4	NA	NaN	NaN	NaN	NaN	NaN	-2.78	0.045
Rhoa	Transforming protein RhoA	11	20	NA	2	2	3	3	7	-2.60	0.050
Dctn4	Dynactin subunit 4	6	5	NA	2	NaN	NaN	2	2	-2.50	0.047
Cdk5	Cyclin-dependent-like kinase 5	3	3	NA	1	NaN	NaN	2	NaN	-2.49	0.045
Arcn1	Coatomer subunit delta	16	8	1	NaN	NaN	NaN	NaN	3	-3.38	0.048
Mapk8ip3	C-Jun-amino-terminal kinase-interacting protein 3	11	14	NA	NaN	2	NaN	NaN	5	-3.23	0.048
Rab31	Ras-related protein Rab-31	12	6	1	NaN	NaN	NaN	NaN	1	-3.22	0.049
Hip1r	Huntingtin-interacting protein 1-related protein	10	8	NA	NaN	NaN	NaN	3	NaN	-3.13	0.052
Snx18	Sorting nexin	7	11	NA	NaN	1	NaN	NaN	2	-3.10	0.053
Uso1	General vesicular transport factor p115	8	6	NA	1	NaN	NaN	NaN	NaN	-2.91	0.053
Snx3	Sorting nexin-3	6	7	NA	NaN	NaN	NaN	NaN	3	-2.85	0.046
Rab5b	Ras-related protein Rab-5B	5	4	NA	1	1	NaN	NaN	NaN	-2.75	0.054
Sh3glb1	Isoform 2 of Endophilin-B1	4	5	NaN	NaN	NaN	NaN	NaN	NaN	-2.74	0.050
Scfd1	Sec1 family domain-containing protein 1	6	5	NA	NaN	1	NaN	NaN	2	-2.57	0.045
Picalm	Phosphatidylinositol-binding clathrin assembly protein	5	3	NA	NaN	NaN	NaN	NaN	NaN	-2.36	0.046
Srcin1	SRC kinase signaling inhibitor 1	20	13	NA	NaN	NaN	NaN	NaN	NaN	-4.57	0.055
Hnrnpd	Heterogeneous nuclear ribonucleoprotein D0	16	11	NA	NaN	NaN	NaN	NaN	NaN	-4.39	0.053
Ampd3	AMP deaminase 3	9	6	NA	NaN	NaN	NaN	NaN	NaN	-4.01	0.057
Psmc4	26S proteasome regulatory subunit 6B	10	7	NA	3	2	NaN	2	NaN	-4.01	0.046

Supplementary Table 5: Mass spectrometry peptide counts for control vs. iBot;*Cnp*-Cre oligodendrocytes +/- biotinylation (biontyl)

Gene (cont.)	Description (cont.)	control biotinyl - rep1	control biotinyl - rep2	iBot biotinyl - rep1	iBot biotinyl - rep2	iBot biotinyl - rep3	non-biotinyl - rep1	non-biotinyl - rep2	non-biotinyl - rep3	log2(fold enrichment, cont.)	q-value (adjusted p-value)
Nacad	NAC-alpha domain-containing protein 1	10	6	NA	NaN	NaN	NaN	2	NaN	-4.00	0.052
Dip2b	Disco-interacting protein 2 homolog B	23	13	NA	1	3	NaN	1	4	-3.95	0.054
Srp68	Signal recognition particle subunit SRP68	14	8	NA	2	NaN	NaN	NaN	1	-3.93	0.048
Ptbp1	Polypyrimidine tract-binding protein 1	19	17	NA	NaN	NaN	NaN	NaN	4	-3.79	0.055
Map1a	Microtubule-associated protein 1A	57	39	4	2	2	NaN	7	13	-3.77	0.044
Qki	Isoform 6 of Protein quaking	20	36	7	NaN	NaN	NaN	NaN	NaN	-3.67	0.057
Ckap5	Cytoskeleton-associated protein 5	13	13	NA	1	2	NaN	4	4	-3.66	0.044
Mapre1	Microtubule-associated protein RP/EB family member 1	12	10	2	1	1	NaN	NaN	NaN	-3.65	0.052
Rps5	40S ribosomal protein S5 (Fragment)	12	7	3	NaN	NaN	NaN	NaN	NaN	-3.60	0.045
Hyou1	Hypoxia up-regulated protein 1	30	14	12	5	4	NaN	NaN	NaN	-3.56	0.050
Prpf19	Pre-mRNA-processing factor 19	11	12	NA	4	2	NaN	NaN	2	-3.55	0.059
Arf5	ADP-ribosylation factor 5	12	6	NA	NaN	2	NaN	NaN	NaN	-3.55	0.045
Atp5pb	ATP synthase F(0) complex subunit B1, mitochondrial	25	14	13	3	NaN	NaN	NaN	NaN	-3.43	0.047
Top1	DNA topoisomerase 1	10	7	NA	NaN	1	NaN	2	1	-3.41	0.053
Acadl	Long-chain-specific acyl-CoA dehydrogenase, mitochondrial	13	7	NA	NaN	NaN	NaN	NaN	4	-3.37	0.048
Dbt	Lipoamide acyltransferase component of branched-chain alpha-keto acid dehydrogenase complex, mitochondrial	22	12	13	NaN	1	NaN	NaN	NaN	-3.37	0.047
Ilf2	Interleukin enhancer-binding factor 2	11	8	1	2	2	NaN	2	NaN	-3.35	0.052
Psmc5	26S proteasome regulatory subunit 8	11	20	NA	1	3	NaN	3	6	-3.34	0.047
Rps27	40S ribosomal protein S27	9	9	NA	NaN	1	NaN	2	NaN	-3.33	0.054
Arhgap35	Rho GTPase-activating protein 35	10	10	NA	1	NaN	NaN	1	5	-3.30	0.046
Ecpas	Proteasome adapter and scaffold protein ECM29	8	5	NA	2	1	NaN	NaN	NaN	-3.25	0.057
Qars1	Glutamine--tRNA ligase	12	8	NA	NaN	4	NaN	NaN	5	-3.24	0.047
Ccar2	Cell cycle and apoptosis regulator protein 2	10	6	2	1	3	NaN	NaN	NaN	-3.18	0.051
Dnmt3a	DNA (cytosine-5)-methyltransferase 3A	7	8	1	1	3	NaN	2	1	-3.16	0.057
Rpl5	60S ribosomal protein L5	23	21	3	2	2	1	2	5	-3.16	0.048
Syncrip	Heterogeneous nuclear ribonucleoprotein Q	14	9	NA	2	5	NaN	6	3	-3.15	0.048
Eif3l	Eukaryotic translation initiation factor 3 subunit L	5	7	NA	NaN	2	NaN	NaN	NaN	-3.14	0.056
Sik3	Serine/threonine-protein kinase SIK3	6	5	NA	NaN	NaN	NaN	NaN	NaN	-3.12	0.056
Ptpn11	Tyrosine-protein phosphatase non-receptor type 11	10	8	NA	1	NaN	NaN	NaN	2	-3.11	0.052
Jmy	Junction-mediating and -regulatory protein	6	3	NA	1	NaN	NaN	1	NaN	-3.08	0.049
Stt3a	Dolichyl-diphosphooligosaccharide--protein glycosyltransferase subunit	9	6	NA	NaN	1	NaN	NaN	1	-3.07	0.045
Eef1b	Elongation factor 1-beta	5	6	NA	NaN	NaN	NaN	1	NaN	-3.07	0.058
Usp14	Ubiquitin carboxyl-terminal hydrolase 14	4	5	NA	NaN	1	NaN	NaN	NaN	-3.06	0.047
Erp44	Endoplasmic reticulum resident protein 44	5	4	NA	NaN	NaN	NaN	NaN	NaN	-3.04	0.053
Bzw1	Basic leucine zipper and W2 domain-containing protein 1	7	7	NA	NaN	NaN	NaN	NaN	NaN	-3.04	0.055
Ssb	Lupus La protein homolog	6	4	NA	NaN	NaN	NaN	NaN	NaN	-3.02	0.049

Supplementary Table 5 (continued)

Gene (cont.)	Description (cont.)	control biotinyl - rep1	control biotinyl - rep2	iBot biotinyl - rep1	iBot biotinyl - rep2	iBot biotinyl - rep3	non-biotinyl - rep1	non-biotinyl - rep2	non-biotinyl - rep3	log2(fold enrichment, cont.)	q-value (adjusted p-value)
Dynlt1	Dynein light chain Tctex-type 1	4	6	NA	2	NaN	NaN	1	NaN	-2.97	0.046
Sh3gl1	Endophilin-A2	4	4	NA	NaN	NaN	NaN	NaN	NaN	-2.97	0.051
Vdac3	Voltage-dependent anion-selective channel protein 3	19	23	4	5	3	1	2	5	-2.95	0.048
Alg2	Alpha-1,3/1,6-mannosyltransferase ALG2	7	6	NA	NaN	NaN	NaN	NaN	1	-2.94	0.055
Abhd12	Lysophosphatidylserine lipase ABHD12	9	16	NA	NaN	2	NaN	NaN	7	-2.91	0.046
Agpat4	1-acyl-sn-glycerol-3-phosphate acyltransferase delta	10	9	NA	3	2	1	3	5	-2.89	0.046
Rpl36	60S ribosomal protein L36	8	6	NA	NaN	NaN	NaN	NaN	NaN	-2.89	0.044
Rpl30	60S ribosomal protein L30	4	6	NA	NaN	2	NaN	NaN	NaN	-2.84	0.049
Tra2a	Transformer-2 protein homolog alpha	4	4	NA	NaN	NaN	NaN	NaN	NaN	-2.84	0.046
Hectd4	HECT domain E3 ubiquitin protein ligase 4	3	4	NA	NaN	NaN	NaN	NaN	NaN	-2.83	0.044
Pds5b	Sister chromatid cohesion protein PDS5 homolog B	4	3	NA	NaN	NaN	NaN	NaN	NaN	-2.83	0.054
Eif3b	Eukaryotic translation initiation factor 3 subunit B	12	6	NA	1	1	NaN	NaN	4	-2.81	0.047
Ppp1r21	Protein phosphatase 1 regulatory subunit 21	7	5	NA	NaN	NaN	NaN	NaN	NaN	-2.80	0.047
Slc25a4	ADP/ATP translocase 1	54	75	10	5	3	16	30	5	-2.78	0.047
Phldb1	Pleckstrin homology-like domain family B member 1	8	4	NA	1	NaN	NaN	NaN	1	-2.72	0.047
Mtch1	Mitochondrial carrier homolog 1	5	3	NA	NaN	NaN	NaN	NaN	NaN	-2.72	0.046
Plxnb3	Plexin-B3	6	10	NA	2	2	NaN	NaN	3	-2.70	0.047
Farsa	Phenylalanine--tRNA ligase alpha subunit	6	5	NA	1	1	NaN	NaN	2	-2.69	0.045
Slc25a18	Mitochondrial glutamate carrier 2	8	9	NA	NaN	NaN	NaN	1	2	-2.66	0.047
Tomm34	Mitochondrial import receptor subunit TOM34	7	4	NA	NaN	NaN	NaN	NaN	NaN	-2.65	0.049
Pcyt2	Ethanolamine-phosphate cytidylyltransferase	5	4	NA	NaN	NaN	NaN	NaN	NaN	-2.64	0.048
Rpl27a	60S ribosomal protein L27a	8	13	NA	2	2	2	3	4	-2.63	0.047
Gcn1	eIF-2-alpha kinase activator GCN1	23	19	5	2	5	NaN	2	6	-2.63	0.046
Mvk	Mevalonate kinase	5	3	NA	NaN	NaN	NaN	NaN	NaN	-2.62	0.046
Tubb3	Tubulin beta-3 chain	4	7	NA	2	NaN	NaN	3	3	-2.61	0.049
Cyp20a1	Cytochrome P450 20A1	4	3	NA	NaN	NaN	NaN	2	NaN	-2.60	0.046
Birc6	Baculoviral IAP repeat-containing protein 6	7	4	NA	NaN	NaN	NaN	NaN	NaN	-2.56	0.049
Rdh11	Retinol dehydrogenase 11	6	8	3	3	2	NaN	3	1	-2.54	0.047
Rpl24	60S ribosomal protein L24	14	8	3	2	3	1	2	2	-2.54	0.047
Sbf1	Myotubularin-related protein 5	8	4	NA	NaN	NaN	NaN	NaN	3	-2.53	0.049
Sec23ip	SEC23-interacting protein	5	4	NA	NaN	NaN	NaN	NaN	NaN	-2.52	0.048
Ppme1	Protein phosphatase methylesterase 1	4	4	NA	NaN	1	NaN	NaN	1	-2.52	0.048
Sec63	Translocation protein SEC63 homolog	4	7	NA	NaN	NaN	NaN	NaN	NaN	-2.51	0.046
Hsdl2	Hydroxysteroid dehydrogenase-like protein 2	6	5	NA	NaN	NaN	NaN	NaN	2	-2.49	0.047
Slc14a1	Urea transporter 1	5	4	NA	NaN	1	NaN	NaN	NaN	-2.48	0.047
Il1rap	Isoform 3 of Interleukin-1 receptor accessory protein	4	4	NA	NaN	NaN	NaN	NaN	NaN	-2.42	0.045
Arpc1b	Actin-related protein 2/3 complex subunit 1B	5	5	1	2	NaN	NaN	2	1	-2.42	0.047
Gltp	Glycolipid transfer protein	4	4	NA	NaN	NaN	1	NaN	1	-2.37	0.046

Supplementary Table 5 (continued)

Gene (cont.)	Description (cont.)	control biotinyl - rep1	control biotinyl - rep2	iBot biotinyl - rep1	iBot biotinyl - rep2	iBot biotinyl - rep3	non-biotinyl - rep1	non-biotinyl - rep2	non-biotinyl - rep3	log2(fold enrichment, cont.)	q-value (adjusted p-value)
Prkcq	Protein kinase C theta type	5	3	NA	NaN	NaN	NaN	NaN	NaN	-2.32	0.049
Arl2	ADP-ribosylation factor-like protein 2	4	3	NA	NaN	NaN	NaN	NaN	NaN	-2.28	0.047
Ranbp3	Ran-binding protein 3	2	2	NA	NaN	NaN	NaN	NaN	NaN	-2.25	0.047
Eif4a2	Eukaryotic initiation factor 4A-II	5	4	NA	2	NaN	NaN	1	2	-2.22	0.049
Arpc4	Actin-related protein 2/3 complex subunit 4	10	6	2	2	2	NaN	2	2	-2.21	0.049
Atxn2	Ataxin-2	4	3	NA	NaN	NaN	NaN	NaN	NaN	-2.21	0.045
Mapt	Microtubule-associated protein tau	3	3	1	NaN	NaN	NaN	1	NaN	-2.20	0.047
Dstn	Destrin	3	3	NA	NaN	NaN	NaN	NaN	NaN	-2.19	0.046
Ddx19a	ATP-dependent RNA helicase DDX19A	6	5	NA	NaN	NaN	NaN	NaN	NaN	-2.09	0.046
Ahcy	Adenosylhomocysteinase	4	3	NA	NaN	NaN	NaN	NaN	1	-2.09	0.049
Eif3h	Eukaryotic translation initiation factor 3 subunit H	3	4	NA	NaN	1	NaN	NaN	1	-2.07	0.049
Agpat1	1-acyl-sn-glycerol-3-phosphate acyltransferase alpha	4	3	NA	NaN	NaN	NaN	NaN	1	-2.07	0.049
Gnpat	Dihydroxyacetone phosphate acyltransferase	4	3	NA	NaN	NaN	NaN	NaN	1	-2.06	0.049
Auh	Methylglutaconyl-CoA hydratase, mitochondrial	3	3	NA	NaN	NaN	NaN	1	1	-2.02	0.049

Supplementary Table 5 (continued)

	Gene	Log_2 fold change (Ribo-seq of remyelination)	$-Log_2$ fold depletion iBot vs control mass spectrometry
1	Mag	2.72	2.98
2	Mbp	2.62	4.05
3	Tppp	1.80	3.01
4	Nacad	1.75	4.00
5	Agpat4	1.72	2.89
6	Plxnb3	1.52	2.70
7	Phldb1	1.44	2.72
8	Sbf1	1.43	2.53
9	Prkcq	1.43	2.32
10	Ank3	1.41	3.82
11	Srcin1	1.37	4.57
12	Bin1	1.36	2.90
13	Pcyt2	1.32	2.64
14	Gltp	1.30	2.37
15	Mapt	1.21	2.20
16	Mvk	1.10	2.62
17	Il1rap	0.98	2.42
18	Nfasc	0.97	4.90
19	Arl2	0.93	2.28
20	Rdh11	0.87	2.54
21	Hip1r	0.77	3.13
22	Ptpn11	0.70	3.11
23	Dip2b	0.68	3.95
24	Ppp1r21	0.66	2.80
25	Rtn4	0.66	3.18
26	Rab31	0.66	3.22
27	Dst	0.62	4.70
28	Dnmt3a	0.59	3.16
29	Cdk5	0.56	2.49
30	Sh3glb1	0.53	2.74
31	Sept8	0.53	2.81
32	Auh	0.49	2.02
33	Bzw1	0.47	3.04
34	Ilf2	0.46	3.35
35	Ptbp1	-0.66	3.79
36	Slc14a1	-0.83	2.48

Supplementary Table 6: Mass spectrometry hits with differential gene expression from Ribo-seq of a mouse model of remyelination (Voskuhl *et al. PNAS* 2019)

Supplementary Figure 14: Source data for Supplementary Fig. 12a

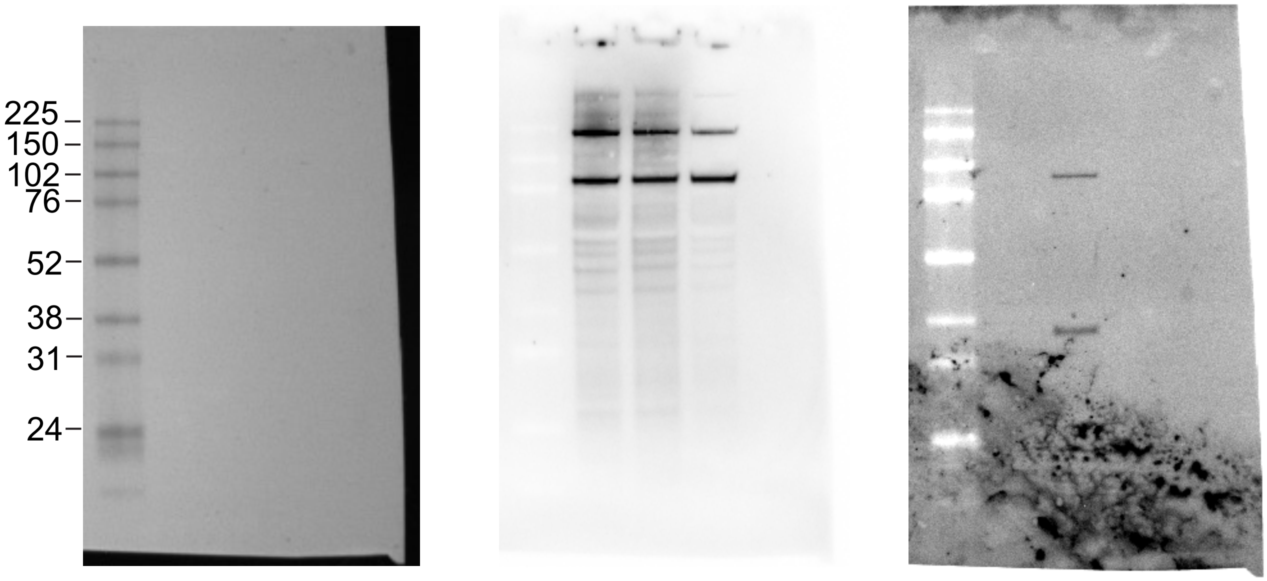

Ladder with molecular weight markers

anti-streptavidin-HRP (1:10000, ThermoFisher S-911)

anti-GFP conjugated to AlexaFluor488 (1:1000, Santa Cruz sc-9996 AF488)

Supplementary References

1. Zhang, Y. *et al.* An RNA-sequencing transcriptome and splicing database of glia, neurons, and vascular cells of the cerebral cortex. *Journal of Neuroscience* **34**, 11929–11947 (2014).
2. Marques, S. *et al.* Oligodendrocyte heterogeneity in the mouse juvenile and adult central nervous system. *Science* **352**, 1326–1329 (2016).
3. Ximerakis, M. *et al.* Single-cell transcriptomic profiling of the aging mouse brain. *Nature neuroscience* **22**, 1696–1708 (2019).
4. Voskuhl, R. R. *et al.* Gene expression in oligodendrocytes during remyelination reveals cholesterol homeostasis as a therapeutic target in multiple sclerosis. *Proceedings of the National Academy of Sciences of the United States of America* **116**, 10130–10139 (2019).
5. Jäkel, S. *et al.* Altered human oligodendrocyte heterogeneity in multiple sclerosis. *Nature* **566**, 543–547 (2019).
