# Supplementary material for: CNS myelination requires VAMP2/3-mediated membrane expansion in oligodendrocytes
Source: Nat Commun. 2022 Sep 23;13:5583. doi: 10.1038/s41467-022-33200-4 (PMC9508103; doi:10.1038/s41467-022-33200-4)
Supplement: Supplementary file 3 — Description of Additional Supplementary Files [file 41467_2022_33200_MOESM3_ESM.pdf]

Description of Additional Supplementary Files

Supplementary Video 1: Exocytosis events in a cultured pre-myelinating oligodendrocyte expressing VAMP2-pHluorin (see Fig. 2b).

Time-lapse imaging of a cultured oligodendrocyte 3 days after differentiation in culture transfected with VAMP2-pHluorin. VAMP2 exocytosis appear as transient fluorescent puncta (batlowK color map). Time in seconds.

Supplementary Video 2: Exocytosis events within myelin sheaths of a zebrafish expressing oligodendrocyte-targeted VAMP2-pHluorin (see Fig. 2d).

Time-lapse imaging of an oligodendrocyte expressing VAMP2-pHluorin in a larval zebrafish spinal cord. Time min:seconds.

Supplementary Video 3: Airyscan confocal z-stack of a myelinated axon (MBP staining) from a P12 iBot;*Cnp*-Cre mouse spinal cord cross section, showing bulges and accumulated vesicles at z = 3400 nm (see Fig. 5b).

Confocal imaging of MBP signal from immunohistochemistry of P12 iBot;*Cnp*-Cre mouse spinal cord with Airyscan-optimized imaging parameters for super-resolution reconstruction.

Title: Supplementary Video 4
Description: Airyscan confocal z-stack of a myelinated axon (MBP staining) from a P12 control littermate mouse spinal cord cross section (Related to Fig. 5b).

Confocal imaging of MBP signal from immunohistochemistry of P12 littermate control mouse spinal cord with Airyscan-optimized imaging parameters for super-resolution reconstruction.
