# Supplementary material for: CNS myelination requires VAMP2/3-mediated membrane expansion in oligodendrocytes
Source: Nat Commun. 2022 Sep 23;13:5583. doi: 10.1038/s41467-022-33200-4 (PMC9508103; doi:10.1038/s41467-022-33200-4)
Supplement: Supplementary file 8 — Reporting Summary [file 41467_2022_33200_MOESM8_ESM.pdf]

nature portfolio

Corresponding author(s): J. Bradley Zuchero

Last updated by author(s): 8/20/2022

Reporting Summary

Nature Portfolio wishes to improve the reproducibility of the work that we publish. This form provides structure for consistency and transparency in reporting. For further information on Nature Portfolio policies, see our Editorial Policies and the Editorial Policy Checklist.

Statistics

For all statistical analyses, confirm that the following items are present in the figure legend, table legend, main text, or Methods section.

n/a	Confirmed	
☐	☒	The exact sample size (n) for each experimental group/condition, given as a discrete number and unit of measurement
☐	☒	A statement on whether measurements were taken from distinct samples or whether the same sample was measured repeatedly
☐	☒	The statistical test(s) used AND whether they are one- or two-sided<br>*Only common tests should be described solely by name; describe more complex techniques in the Methods section.*
☐	☒	A description of all covariates tested
☐	☒	A description of any assumptions or corrections, such as tests of normality and adjustment for multiple comparisons
☐	☒	A full description of the statistical parameters including central tendency (e.g. means) or other basic estimates (e.g. regression coefficient) AND variation (e.g. standard deviation) or associated estimates of uncertainty (e.g. confidence intervals)
☐	☒	For null hypothesis testing, the test statistic (e.g. F, t, r) with confidence intervals, effect sizes, degrees of freedom and P value noted<br>*Give P values as exact values whenever suitable.*
☒	☐	For Bayesian analysis, information on the choice of priors and Markov chain Monte Carlo settings
☒	☐	For hierarchical and complex designs, identification of the appropriate level for tests and full reporting of outcomes
☒	☐	Estimates of effect sizes (e.g. Cohen's d, Pearson's r), indicating how they were calculated

Our web collection on statistics for biologists contains articles on many of the points above.

Software and code

Policy information about availability of computer code

Data collection	Zen Zeiss 2.6 (Blue edition), IncuCyte ZOOM (Essen Biosciences), Orbitrap Q Exactive HF-X (Thermo Scientific)
Data analysis	Fiji/Image J 2.3.0/1.53q (NIH), GraphPad Prism 9.10 (216), Excel 16.63.1 (Microsoft), MatLab R2021b, RStudio 2021.09.0 (R software), Byonic v4.1.5 (Protein Metrics), Cytoscape 3.9.0 (https://cytoscape.org/), Perseus v1.6.5.0 (MaxQuant, https://maxquant.net/perseus/), gProfiler (https://biit.cs.ut.ee/gprofiler/gost)

For manuscripts utilizing custom algorithms or software that are central to the research but not yet described in published literature, software must be made available to editors and reviewers. We strongly encourage code deposition in a community repository (e.g. GitHub). See the Nature Portfolio guidelines for submitting code & software for further information.

Data

Policy information about availability of data

All manuscripts must include a data availability statement. This statement should provide the following information, where applicable:

- Accession codes, unique identifiers, or web links for publicly available datasets
- A description of any restrictions on data availability
- For clinical datasets or third party data, please ensure that the statement adheres to our policy

The data generated in this study are provided in the Supplementary Information and Source Data files. Source data are provided with this paper. The mass spectrometry proteomics data have been deposited to the ProteomeXchange Consortium via the PRIDE partner repository with the dataset identifier PXD036174 and 10.6019/PXD036174. Step-by-step protocols are available from the corresponding or first authors upon request. All DNA constructs created in this study have been deposited at Addgene: AAV_pCMV-Vamp2-pHluorin (Addgene plasmid 190151), AAV_pCMV-Vamp3-pHluorin (Addgene plasmid 190152), AAV_pMBP-dnVamp2-P2AT2A-EGFP-caax (Addgene plasmid 190153), AAV_pMBP-dnVamp3-P2AT2A-EGFP-caax (Addgene plasmid 190154), AAV_pMBP-EGFP-caax (Addgene

plasmid 190155). Correspondence and requests for all other materials should be addressed to M.L or J.B.Z.

Field-specific reporting

Please select the one below that is the best fit for your research. If you are not sure, read the appropriate sections before making your selection.

[x] Life sciences [ ] Behavioural & social sciences [ ] Ecological, evolutionary & environmental sciences

For a reference copy of the document with all sections, see nature.com/documents/nr-reporting-summary-flat.pdf

Life sciences study design

All studies must disclose on these points even when the disclosure is negative.

Sample size	Sample sizes were chosen based on Zuchero et al. 2015 Dev. Cell (PMID: 26166300), where power analyses determined that n = 3-5 mice per genotype for tissue and n = 3 biological replicates for culture experiments are required for 90-99% power with an alpha level of 0.01. For live cell imaging of exocytosis, we used n = 12-33 cells per biological replicate based on Urbina et al. 2018 JCB (PMID: 29351997). For zebrafish studies, our sample size was based on Almeida et al. 2021 Curr Biol. (PMID: 34270947).
Data exclusions	For imaging of exocytosis events, cells were excluded if they exhibited low baseline GFP fluorescence that matched untransfected cells or if they had discontinuous, broken morphology arising from cell stress. For mass spectrometry, common contaminants, such as keratin and titin as classified by the Byonic software, were excluded from downstream analysis, and one biological replicate was excluded due to an absence of peptide counts.
Replication	All biological replicates are reported in the figure legends. CNS tissues for immunohistochemistry and EM analysis were collected from 4-5 different animals of each genotype. PNS tissues were collected from 3 animals of each genotype. Each biological replicate for cell images represents the mean of all datapoints harvested from a single mouse brain. Cells from 4-5 different brains of each genotype were analyzed for monoculture and co-culture experiments. Oligodendrocytes for mass spectrometry were harvested from 5 control littermates (2 biotinylated, 3 non-biotinylated) and 3 iBot;Cnp-Cre mice.
Randomization	Mice of the same genotype were randomly selected for experiments involving immunohistochemistry, electron microscopy, viability, and primary oligodendrocyte purification. Rat pups were randomly selected for primary oligodendrocyte and retinal ganglion cell purification. Zebrafish were screened for transgene expression and then randomly selected for imaging.
Blinding	Image acquisition and analysis for electron microscopy, immunohistochemistry, and cellular assays (including live cell imaging and co-cultures) were performed with the researcher blinded to the genotype. For proteomics, sample collection and immunoprecipitation was not blinded to minimize the chance of mixing up samples. Blinding for the zebrafish imaging was not relevant because the cells were classified based on morphology.

Reporting for specific materials, systems and methods

We require information from authors about some types of materials, experimental systems and methods used in many studies. Here, indicate whether each material, system or method listed is relevant to your study. If you are not sure if a list item applies to your research, read the appropriate section before selecting a response.

Materials & experimental systems

n/a	Involved in the study	
[ ]	[x]	Antibodies
[ ]	[x]	Eukaryotic cell lines
[x]	[ ]	Palaeontology and archaeology
[ ]	[x]	Animals and other organisms
[x]	[ ]	Human research participants
[x]	[ ]	Clinical data
[x]	[ ]	Dual use research of concern

Methods

n/a	Involved in the study	
[x]	[ ]	ChIP-seq
[x]	[ ]	Flow cytometry
[x]	[ ]	MRI-based neuroimaging

Antibodies

Antibodies used

1) rat anti-MBP (Abcam ab7349, 1:100 for tissue, 1:400 for cultured cells)
2) rabbit anti-Olig2 (Sigma AB9610, 1:1000)
3) mouse anti-CC1 (Oncogene OP80, 1:100)
4) rabbit anti-Caspr (Abcam ab34151, 1:1000)
5) mouse anti-AnkG (Sigma MABN465, 1:500)
6) mouse anti-NF200 (Sigma N0142, 1:100)
7) chicken anti-GFP (Abcam 13970, 1:1000)
8) mouse-anti MAG clone 513 (EMD Millipore MAB1567, 1:20 for surface staining, 1:100 for total MAG)

9) mouse anti-galactosylceramide (GalCer) hybridoma (Emery and Dugas, 2013, Source: Dr. Barbara Ranscht, PMID: 7045870, 1:50).

The following secondary antibodies were used at a 1:1000 dilution for tissue and cell staining: donkey anti-rat Alexa Fluor 594 (Thermo Scientific A-21209), goat anti-rat Alexa Fluor 647 (Thermo Scientific A-21247), donkey anti-rabbit Alexa Fluor 647 (Thermo Scientific A-31573), donkey anti-mouse Alexa Fluor 594 (Thermo Scientific A-21203), donkey anti-mouse Alexa Fluor 647 (Thermo Scientific A-31571), and goat anti-chicken Alexa Fluor 488 (Thermo Scientific A-32931).

Validation

1) Mbp is knockout validated in Zuchero, J. B. et al. 2015 Dev. Cell (PMID: 26166300)
2) anti-Olig2 (Sigma AB9610) was shown to be specific for oligodendrocyte-lineage cells by IHC in Gautier et al 2015 Nat Comm (PMID: 26439639)
3) anti-CC1 (Oncogene OP80) was found to recognize Quaking 7, an RNA-binding protein that is highly upregulated in oligodendrocytes, in Bin et al 2016 J. Neurochem (PMID: 27454326)
4) anti-Caspr (Abcam ab34151) was knockout validated by Western blotting of Caspr KO HAP1 whole cell lysate (https://www.abcam.com/caspr-antibody-ab34151.html#lb)
5) anti-AnkG (Sigma MABN465) was used in Zhou et al 2021 eLife (PMID: 33942715) to detect nodes of Ranvier.
6) anti-NF200: https://www.sigmaaldrich.com/US/en/product/sigma/n0142
7) anti-GFP (Abcam 13970) was appeared specifically in the iBot;Cnp-Cre samples by IHC and Western blotting within this manuscript.
8) anti-MAG: https://www.emdmillipore.com/US/en/product/Anti-Myelin-Associated-Glycoprotein-Antibody-clone-513,MM_NF-MAB1567
9) anti-galactosylceramide (GalCer) hybridoma was shown to specifically recognize differentiating oligodendrocytes and not progenitors in Emery and Dugas, CSH Protocol 2013 (PMID: 29093196).

Eukaryotic cell lines

Policy information about cell lines

Cell line source(s)	Primary cultures of oligodendrocyte precursors and retinal ganglion cells were prepared using Sprague-Dawley rats and C57BL/6 mice (ordered from Charles River) using previously published protocols.
Authentication	Primary oligodendrocytes were authenticated by staining for galactosylceramide lipid (1:50 hybridoma) and MBP (Abcam ab7349 1:100). Retinal ganglion cells were authenticated with expression of Neurofilament (Sigma, N4142, lot #037M4872V, 1:1000).
Mycoplasma contamination	Primary cultures were acutely purified from mice/rats, and were not tested for mycoplasma contamination. We did not conduct any experiments with immortalized cell lines.
Commonly misidentified lines (See ICLAC register)	Commonly misidentified lines were not used.

Animals and other organisms

Policy information about studies involving animals; ARRIVE guidelines recommended for reporting animal research

Laboratory animals

Mice were group-housed under standard 12:12 light-dark cycles at temperatures of 18-23 degrees Celsius and 40-60% humidity with free access to food and water, and disposable bedding in plastic cages (APLAC Protocol 32260).
Cnp-CRE/+ mice were obtained from Dr. Klaus Nave (Max Planck Institute for Experimental Medicine) and maintained through crossing to C57BL/6 mice. iBot mice were obtained from Dr. Frank Pfrieger (University of Strasbourg) and Dr. Shawn Hentges (Colorado State University). Transgenic mice used for IHC and EM were harvested at post-natal day 12. Transgenic mice for primary oligodendrocyte isolation were harvested at postnatal day 6-7. Males and females were pooled for analysis because no sexual dimorphism for myelination defects were observed after the conditional expression of iBot. The sexes of each mouse sample are indicated by a square or circle symbol in the Figures and Supplementary Information throughout the manuscript.
Sprague-Dawley rats and C57BL/6 mice were ordered from Charles River Laboratories and harvested at postnatal day 6 for primary oligodendrocyte isolation or at postnatal day 4-5 for retinal ganglion cell isolation. Cells derived from males and females were pooled for analysis.
Transgenic Tg(olig1:KalTA4) and Tg(claudinK:Gal4) zebrafish were originally obtained from Dr. Tim Czopka and Prof. Thomas Becker respectively, and local colonies maintained through incrossing or outbreeding with wildtype strains. Transgenic larvae were analysed between 4 and 5 days post fertilization, before the onset of sexual differentiation.

Wild animals	The study did not involve wild animals.
Field-collected samples	The study did not involve field-collected samples.
Ethics oversight	All procedures involving animals were approved by the Institutional Administrative Panel on Laboratory Animal Care (APLAC) of Stanford University and followed the National Institutes of Health guidelines.

Note that full information on the approval of the study protocol must also be provided in the manuscript.
